# Supplementary material for: PLATO: A Predictive Drug Discovery Web Platform for Efficient Target Fishing and Bioactivity Profiling of Small Molecules
Source: Int J Mol Sci. 2022 May 8;23(9):5245. doi: 10.3390/ijms23095245 (PMC9103655; doi:10.3390/ijms23095245)
Supplement: Supplementary file 1 [file ijms-23-05245-s001.zip › Bioactivity_Profiling_Case_study_2.pdf]

quantitative bioactivity profiling report

smiles: O=C(Nc1ccc(Cl)cc1)OC(COc2ccccc2)Cn3ccnc3

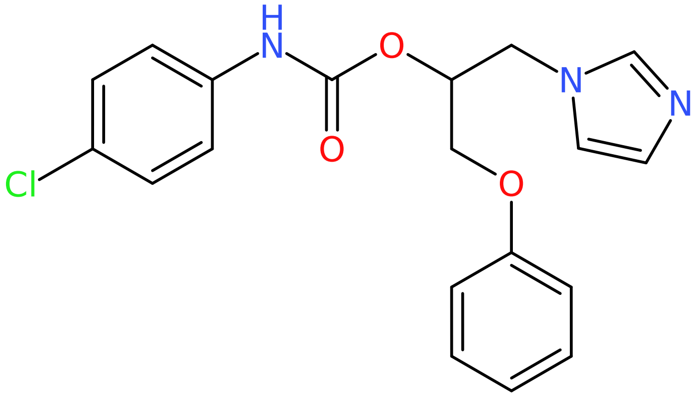

Target	IC50	EC50	Ki	Kd	σ_P
Neuronal acetylcholine receptor protein alpha-7 subunit:Rattus norvegicus	306nM	420nM	192nM	4.90nM	2.1: IC50
Neurokinin 1 receptor:Homo sapiens	13.1nM	22.3nM	8.78nM	9.81nM	2.1: IC50
Orexin receptor 2:Homo sapiens	101nM	145nM	112nM	100nM	2.1: Ki
Orexin receptor 1:Homo sapiens	160nM	3.95μM	305nM	177nM	2.2: Ki
Inosine-5'-monophosphate dehydrogenase:Bacillus anthracis	97.4nM		32.0nM		2.2: IC50
Cytochrome P450 19A1:Homo sapiens	598nM		79.7nM		2.3: IC50
15-cis-phytoene desaturase:Synechococcus elongatus (strain PCC 7942) (Anacystis nidulans R2)	211nM				2.3: IC50
Protein farnesyltransferase:Homo sapiens	118nM	7.69nM	583nM	2.00nM	2.3: IC50
Thromboxane-A synthase:Homo sapiens	129nM			452nM	2.4: IC50
Metabotropic glutamate receptor 2:Homo sapiens	40.3nM	223nM	85.9nM	10.0nM	2.4: EC50
Inosine-5'-monophosphate dehydrogenase, probable:Cryptosporidium parvum	90.3nM	3.30μM	10.9nM		2.4: IC50
Mitochondrial complex V; ATP synthase:Bos taurus	704nM				2.4: IC50
Vascular endothelial growth factor receptor 2:Homo sapiens	151nM	65.2nM	95.6nM	123nM	2.4: IC50
Geranylgeranyl transferase type I:Homo sapiens	883nM	389nM	8.00nM		2.4: IC50
Vascular endothelial growth factor receptor 1:Homo sapiens	255nM	454nM	59.8nM	116nM	2.4: IC50
Histamine H3 receptor:Rattus norvegicus	15.3nM	3.97nM	14.9nM	6.42nM	2.4: Ki
Neuronal acetylcholine receptor protein alpha-7 subunit:Homo sapiens	2.30μM	705nM	207nM	19.3nM	2.4: Ki
Histamine H1 receptor:Cavia porcellus	176nM	111nM	334nM	242nM	2.4: Ki
Histamine H2 receptor:Cavia porcellus	2.15μM	80.5nM	3.77μM	1.16μM	2.4: Ki
Acetylcholine-binding protein:Lymnaea stagnalis			663nM	670nM	2.4: Kd
Human immunodeficiency virus type 1 Tat protein:Human immunodeficiency virus type 1 group M subtype B (isolate HXB2)(HIV-1)	10.6μM	2.24μM		3.72μM	2.5: EC50
Coagulation factor X:Homo sapiens	77.6nM		70.9nM	33.2nM	2.5: Ki

Target	IC50	EC50	Ki	Kd	σ_P
Liver glycogen phosphorylase:Homo sapiens	296nM		3.35µM	2.12µM	2.5: IC50
Coagulation factor IX:Homo sapiens	203nM		34.9nM		2.5: Ki
Urokinase-type plasminogen activator:Homo sapiens	1.62µM		977nM		2.5: Ki
Dopamine transporter:Rattus norvegicus	116nM	771nM	150nM	4.65nM	2.5: Ki
Anandamide amidohydrolase:Rattus norvegicus	246nM	212nM	36.0nM	0.60nM	2.5: IC50
Heme oxygenase 1:Rattus norvegicus	4.03µM				2.5: IC50
Human immunodeficiency virus type 1 reverse transcriptase:Human immunodeficiency virus 1	653nM	339nM	212nM	10.5µM	2.5: IC50
Prostanoid EP1 receptor:Homo sapiens	36.7nM	277nM	291nM	60.4nM	2.5: IC50

tid: 12167

chembl id: CHEMBL4980

name: Neuronal acetylcholine receptor protein alpha-7 subunit:Rattus norvegicus

similarity analysis

structure	chembl id	τ	IC50	EC50	Ki	Kd
global result		0.044: IC50	306nM	420nM	192nM	4.90nM
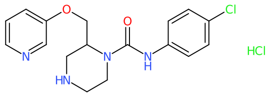	CHEMBL3262153	0.024	70.0nM			
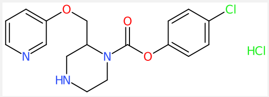	CHEMBL3262155	0.024	9.50nM			
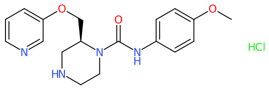	CHEMBL3262164	0.018	18.0μM			
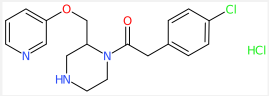	CHEMBL3262150	0.018	110nM			
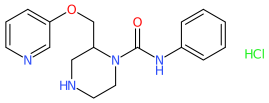	CHEMBL3262152	0.018	680nM			
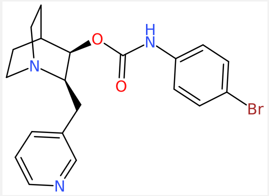	CHEMBL371517	0.012			5.00nM	
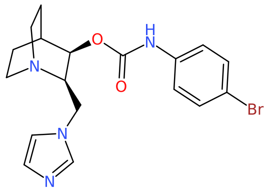	CHEMBL193743	0.012			10.0nM	
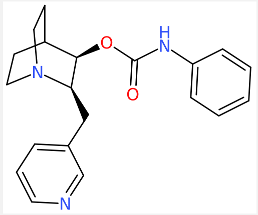	CHEMBL194115	0.012			7.00nM	
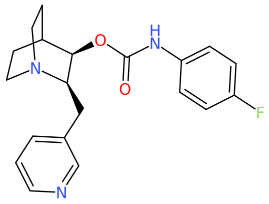	CHEMBL193441	0.012			6.00nM	

structure	chembl id	τ	IC50	EC50	Ki	Kd
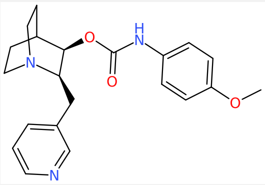	CHEMBL194083	0.012		300nM	5.00nM	
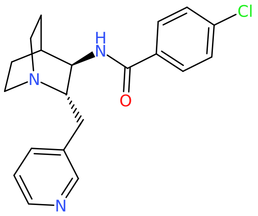	CHEMBL2179842	0.012		200nM		
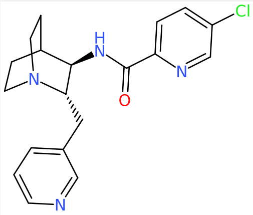	CHEMBL2179857	0.012		270nM		
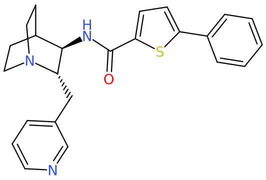	CHEMBL2179875	0.0092		130nM		
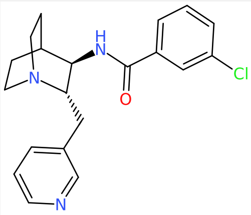	CHEMBL2179841	0.0092		600nM		
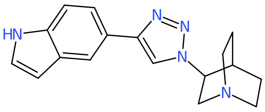	CHEMBL3746799	0.0067				120nM
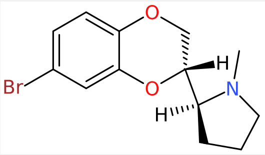	CHEMBL1917228	0.006				0.30nM
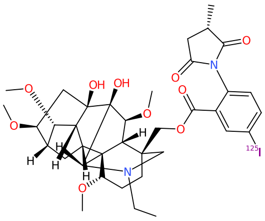	CHEMBL2113668	0.005				1.98nM

tid: 250

chembl id: CHEMBL249

name: Neurokinin 1 receptor:Homo sapiens

similarity analysis

structure	chembl id	τ	IC50	EC50	Ki	Kd
global result		0.044: IC50	13.1nM	22.3nM	8.78nM	9.81nM
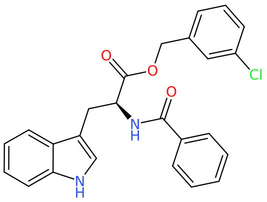	CHEMBL279916	0.024	1.05µM			
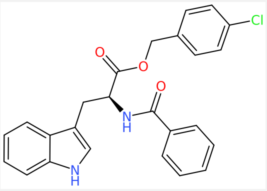	CHEMBL279500	0.024	243nM			
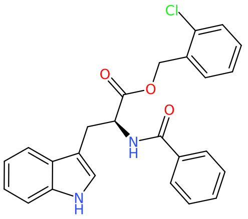	CHEMBL23927	0.018	600nM			
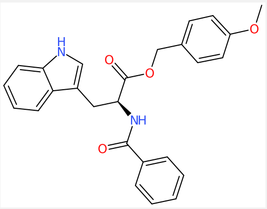	CHEMBL282728	0.018	5.50µM			
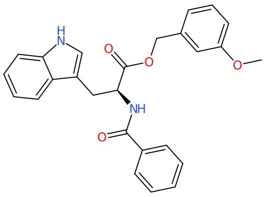	CHEMBL23928	0.018	760nM			
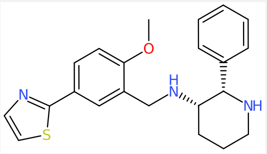	CHEMBL147619	0.0092			0.79nM	
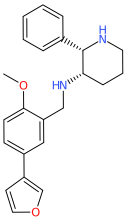	CHEMBL356786	0.0092			0.06nM	

structure	chembl id	τ	IC50	EC50	Ki	Kd
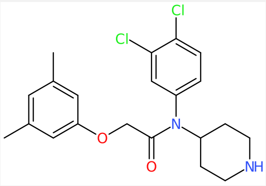	CHEMBL91418	0.0092			25.1nM	
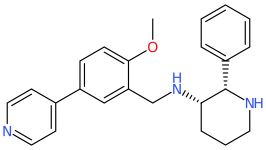	CHEMBL359188	0.0092			0.06nM	
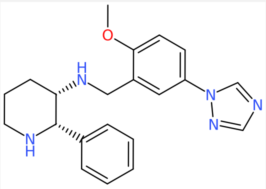	CHEMBL359255	0.0092			0.16nM	
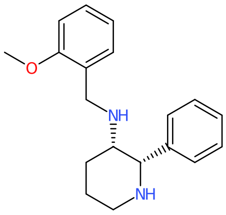	CHEMBL441225	0.0077				1.26nM
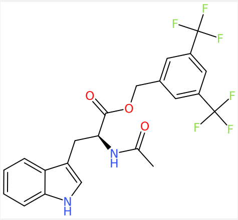	CHEMBL22870	0.0077				3.98nM
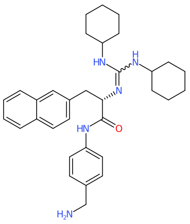	CHEMBL58873	0.0077				1.30µM
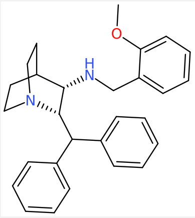	CHEMBL16192	0.0077				2.74nM
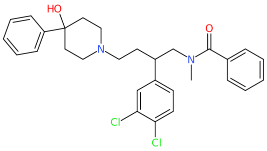	CHEMBL71397	0.0077				0.32nM
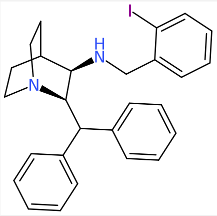	CHEMBL24999	0.0067		24.4nM		
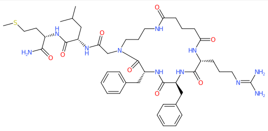	CHEMBL303222	0.005		11.0nM		

structure	chembl id	τ	IC50	EC50	Ki	Kd
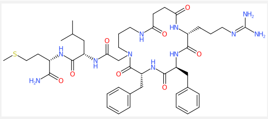	CHEMBL421485	0.005		180nM		
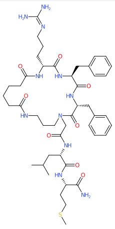	CHEMBL302349	0.005		5.00nM		

tid: 12968

· chembl id: CHEMBL4792

name: Orexin receptor 2:Homo sapiens

similarity analysis

structure	chembl id	τ	IC50	EC50	Ki	Kd
global result		0.041: Ki	101nM	145nM	112nM	100nM
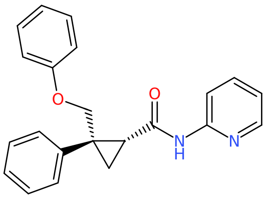	CHEMBL3343247	0.024			967nM	
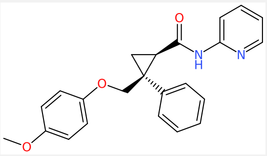	CHEMBL3341773	0.018			588nM	
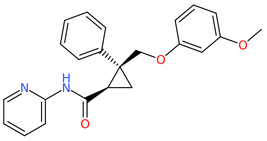	CHEMBL3343249	0.018			1.33μM	
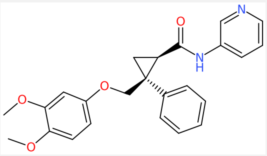	CHEMBL3343252	0.018			51.0nM	
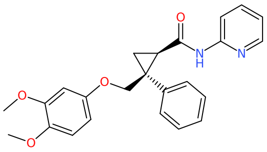	CHEMBL3343246	0.018			36.0nM	
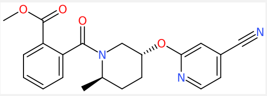	CHEMBL3426151	0.012	21.0nM			
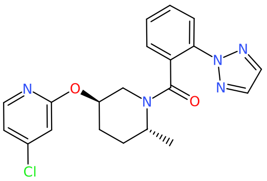	CHEMBL3426141	0.012	11.0nM			
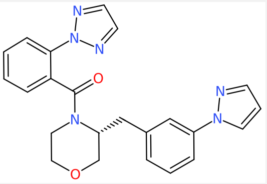	CHEMBL3932552	0.0092	1.82μM			
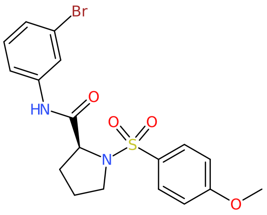	CHEMBL3667567	0.0092	37.0nM			

structure	chembl id	τ	IC50	EC50	Ki	Kd
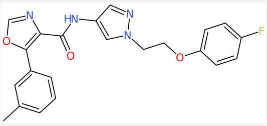	CHEMBL3912238	0.0092	973nM			
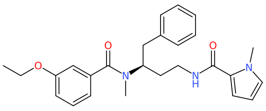	CHEMBL2413517	0.0092				2.00nM
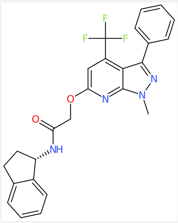	CHEMBL3665672	0.0077				128nM
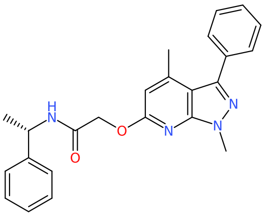	CHEMBL3665674	0.0077				143nM
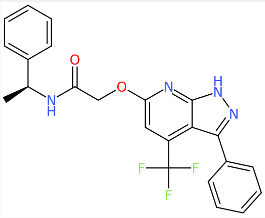	CHEMBL3634019	0.0077				184nM
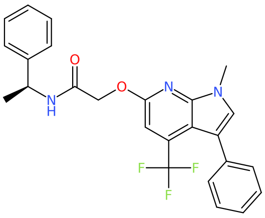	CHEMBL3665733	0.0077				240nM
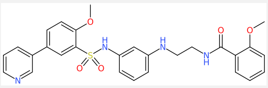	CHEMBL3622426	0.0077		268nM		
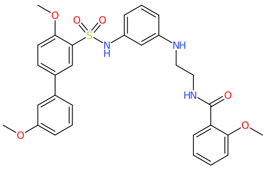	CHEMBL3622419	0.0067		800nM		
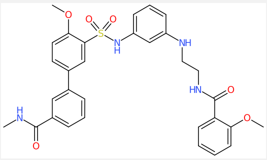	CHEMBL3622421	0.0067		182nM		
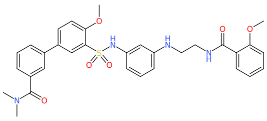	CHEMBL3622422	0.0067		50.0nM		
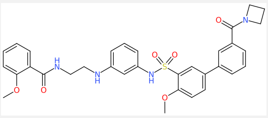	CHEMBL3622423	0.0067		235nM		

tid: 10009

chembl id: CHEMBL5113

name: Orexin receptor 1:Homo sapiens

similarity analysis

**structure**	**chembl id**	***τ***	**IC50**	**EC50**	**Ki**	**Kd**
global result		0.041: Ki	160nM	3.95μM	305nM	177nM
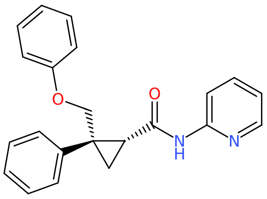	CHEMBL3343247	0.024			6.84μM	
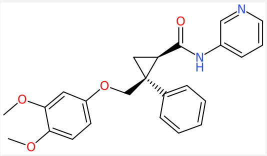	CHEMBL3343252	0.018			206nM	
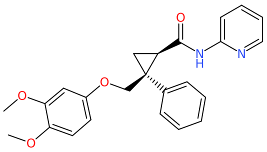	CHEMBL3343246	0.018			1.07μM	
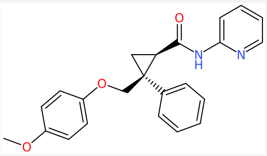	CHEMBL3341773	0.018			5.54μM	
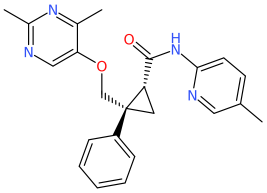	CHEMBL3585949	0.012			124nM	
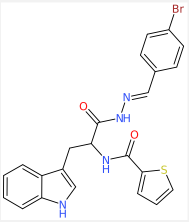	CHEMBL2007173	0.012	4.70μM			
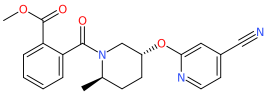	CHEMBL3426151	0.012	10.00μM			
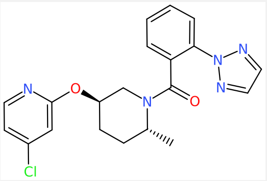	CHEMBL3426141	0.012	550nM			

structure	chembl id	τ	IC50	EC50	Ki	Kd
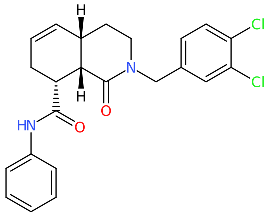	CHEMBL1714761	0.012	1.44µM			
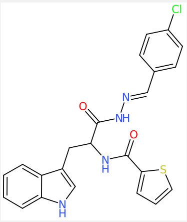	CHEMBL1973904	0.012	5.34µM			
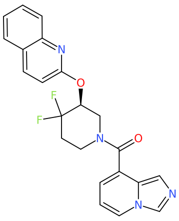	CHEMBL3955752	0.0092				0.94nM
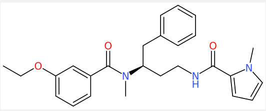	CHEMBL2413517	0.0092				696nM
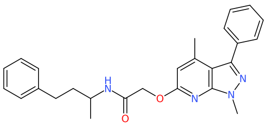	CHEMBL3665675	0.0077				116nM
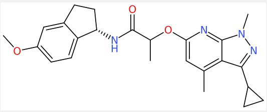	CHEMBL3665732	0.0077				589nM
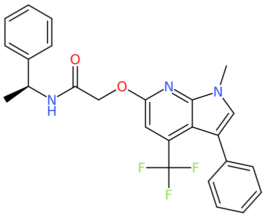	CHEMBL3665733	0.0077				501nM
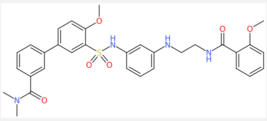	CHEMBL3622422	0.0067		5.23µM		
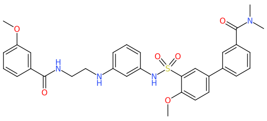	CHEMBL3622428	0.0067		9.25µM		
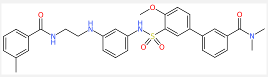	CHEMBL3623075	0.0067		1.62µM		
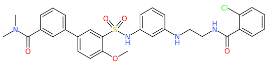	CHEMBL3623076	0.0067		6.13µM		
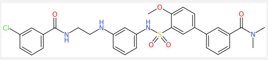	CHEMBL3623077	0.0067		5.72µM		

tid:	109471
chembl id:	CHEMBL3329078
name:	Inosine-5'-monophosphate dehydrogenase:Bacillus anthracis

similarity analysis

structure	chembl id	τ	IC50	EC50	Ki	Kd
global result		0.038: IC50	97.4nM		32.0nM	
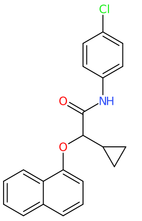	CHEMBL564117	0.024	160nM			
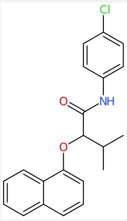	CHEMBL557281	0.018	120nM			
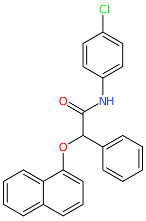	CHEMBL564187	0.012	2.40µM			
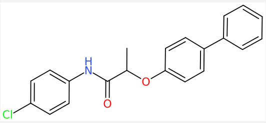	CHEMBL3329559	0.012	150nM			
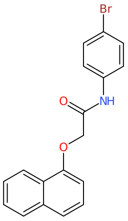	CHEMBL3329560	0.012	760nM			
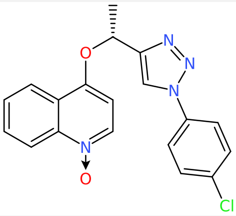	CHEMBL549612	0.0092			46.9nM	
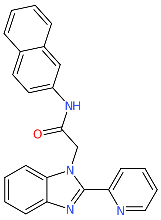	CHEMBL1957258	0.0092			190nM	

structure	chembl id	τ	IC50	EC50	Ki	Kd
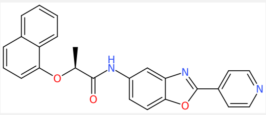	CHEMBL2348823	0.0092			20.2nM	
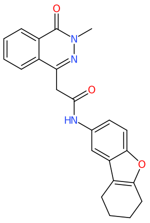	CHEMBL2315283	0.0077			77.5nM	
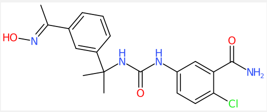	CHEMBL2178617	0.0067			3.46nM	

tid:	65
chembl id:	CHEMBL1978
name:	Cytochrome P450 19A1:Homo sapiens

similarity analysis

structure	chembl id	τ	IC50	EC50	Ki	Kd
global result		0.036: IC50	598nM		79.7nM	
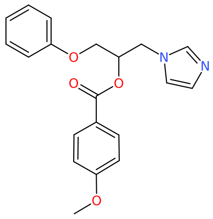	CHEMBL1485809	0.024	16.5nM			
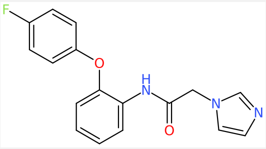	CHEMBL1784798	0.012	248nM			
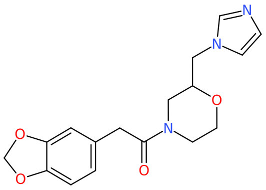	CHEMBL1784812	0.012	46.7nM			
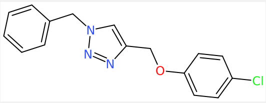	CHEMBL1825044	0.012	5.04µM			
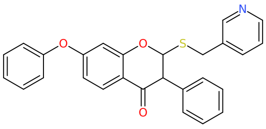	CHEMBL1096744	0.012	210nM			
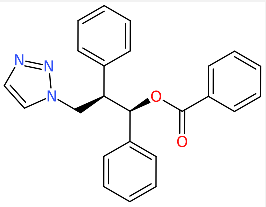	CHEMBL1933693	0.018			100.0nM	
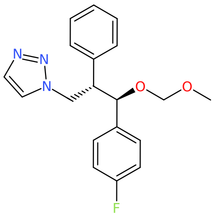	CHEMBL2443356	0.0092			160nM	
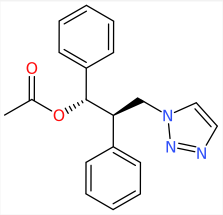	CHEMBL1933701	0.0092			251nM	

structure	chembl id	τ	IC50	EC50	Ki	Kd
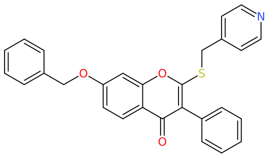	CHEMBL188664	0.0092			220nM	
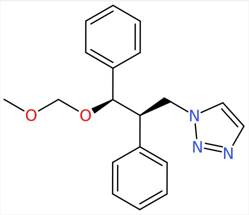	CHEMBL193369[illegible]	0.0092			792nM	

tid:	106616
chembl id:	CHEMBL2366472
name:	15-cis-phytoene desaturase:Synechococcus elongatus (strain PCC 7942) (Anacystis nidulans R2)

similarity analysis

structure	chembl id	τ	IC50	EC50	Ki	Kd
global result		0.035: IC50	211nM			
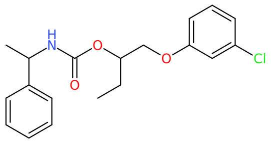	CHEMBL2272706	0.018	63.1nM			
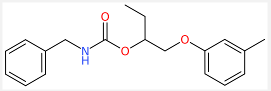	CHEMBL2272992	0.018	126nM			
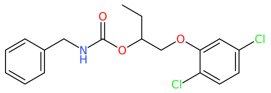	CHEMBL2272970	0.018	3.98µM			
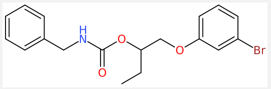	CHEMBL2272989	0.018	79.4nM			
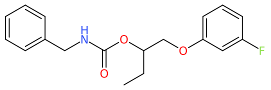	CHEMBL2272988	0.018	63.1nM			

tid:	104690
chembl id:	CHEMBL2094108
name:	Protein farnesyltransferase:Homo sapiens

similarity analysis

structure	chembl id	τ	IC50	EC50	Ki	Kd
global result		0.035: IC50	118nM	7.69nM	583nM	2.00nM
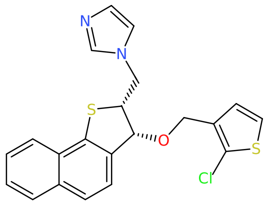	CHEMBL133625	0.018	1.50µM			
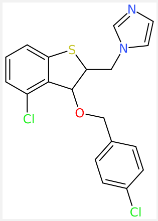	CHEMBL335517	0.018	4.56µM			
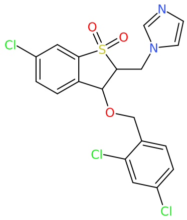	CHEMBL336835	0.018	45.9µM			
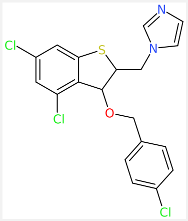	CHEMBL133288	0.018	1.40µM			
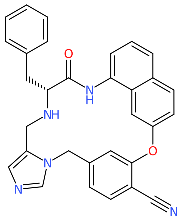	CHEMBL299568	0.012	0.47nM			
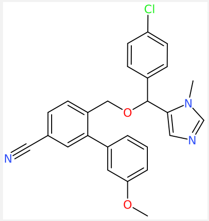	CHEMBL347172	0.0092		17.7nM		
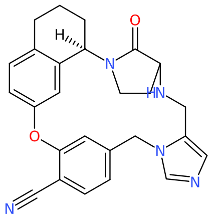	CHEMBL442552	0.0092		0.18nM		

structure	chembl id	τ	IC50	EC50	Ki	Kd
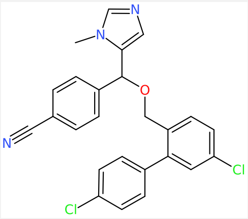	CHEMBL158262	0.0092		525nM		
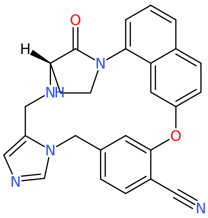	CHEMBL53821	0.0092		79.0nM		
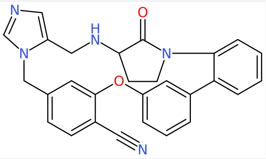	CHEMBL310586	0.0092		0.62nM		
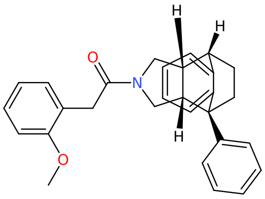	CHEMBL300009	0.0077			40.0µM	
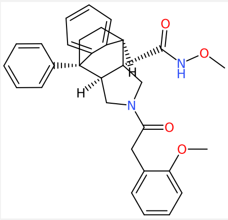	CHEMBL301809	0.0077			420nM	
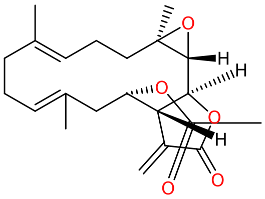	CHEMBL354262	0.0067			170nM	
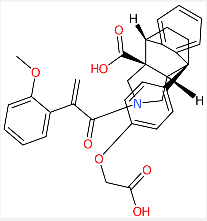	CHEMBL297030	0.0067			25.5µM	
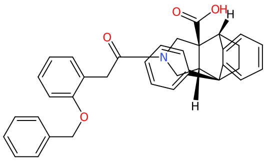	CHEMBL50661	0.0067			32.0µM	
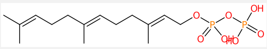	CHEMBL69330	0.005				2.00nM

tid:	248
chembl id:	CHEMBL1835
name:	Thromboxane-A synthase:Homo sapiens

similarity analysis

structure	chembl id	τ	IC50	EC50	Ki	Kd
global result		0.034: IC50	129nM			452nM
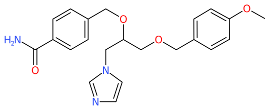	CHEMBL290637	0.018	6.00µM			
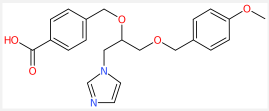	CHEMBL38825	0.018	2.71µM			
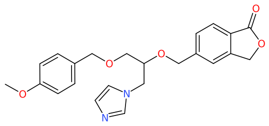	CHEMBL290933	0.018	6.20µM			
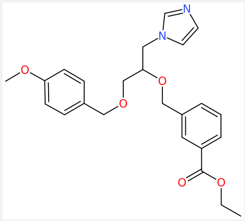	CHEMBL41865	0.012	7.20µM			
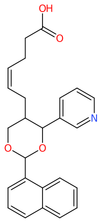	CHEMBL160657	0.012	40.0nM			
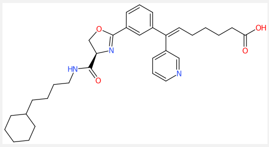	CHEMBL294850	0.0067				452nM

tid: 12895
· chembl id: CHEMBL5137
name: Metabotropic glutamate receptor 2:Homo sapiens

similarity analysis

structure	chembl id	τ	IC50	EC50	Ki	Kd
global result		0.034: EC50	40.3nM	223nM	85.9nM	10.0nM
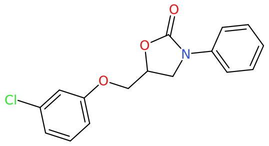	CHEMBL1097984	0.018		840nM		
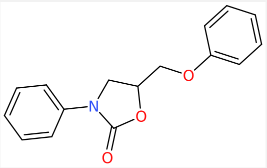	CHEMBL1095705	0.018		3.50µM		
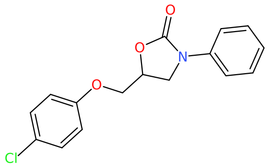	CHEMBL1094733	0.018		360nM		
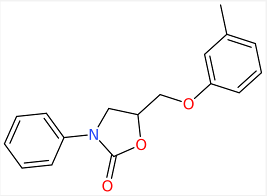	CHEMBL1098388	0.012		3.44µM		
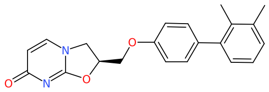	CHEMBL4288731	0.012		330nM		
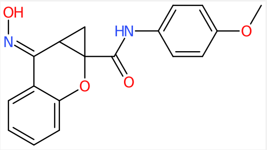	CHEMBL1630807	0.0092	1.00µM		800nM	
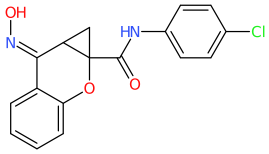	CHEMBL1630805	0.0092	800nM		1.00µM	
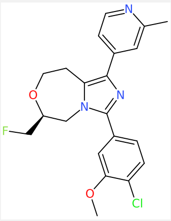	CHEMBL4107888	0.0092	13.5nM			
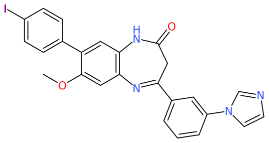	CHEMBL3133887	0.0092	89.0nM			

structure	chembl id	τ	IC50	EC50	Ki	Kd
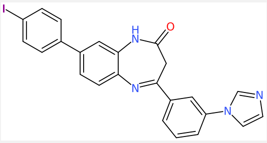	CHEMBL3133884	0.0092	133nM			
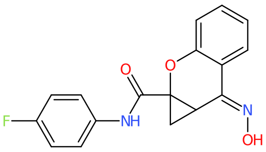	CHEMBL1630804	0.0077			6.60µM	
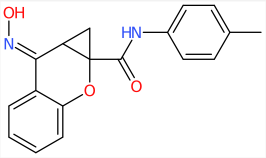	CHEMBL1630806	0.0077			600nM	
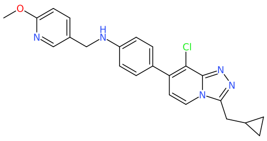	CHEMBL4072134	0.0077			7.41nM	15.6nM
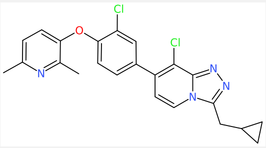	CHEMBL4074664	0.0077				1.00nM
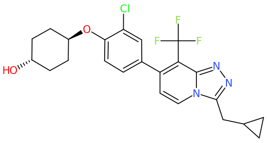	CHEMBL4077609	0.0067				3.00nM
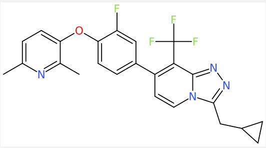	CHEMBL4080973	0.0067				2.50nM
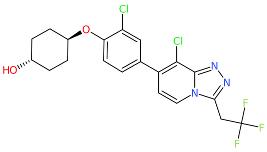	CHEMBL4081244	0.0067				16.6nM

tid:	102448
chembl id:	CHEMBL6145
name:	Inosine-5'-monophosphate dehydrogenase, probable:Cryptosporidium parvum

similarity analysis

structure	chembl id	τ	IC50	EC50	Ki	Kd
global result		0.034: IC50	90.3nM	3.30µM	10.9nM	
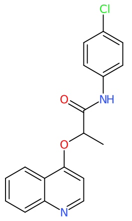	CHEMBL570997	0.018	753nM			
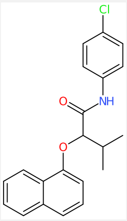	CHEMBL557281	0.018	744nM			
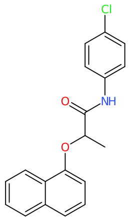	CHEMBL561635	0.018	1.32µM			
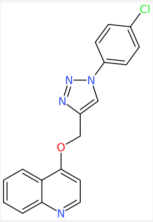	CHEMBL554061	0.012	460nM			
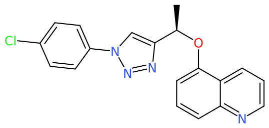	CHEMBL549610	0.012	16.4nM			
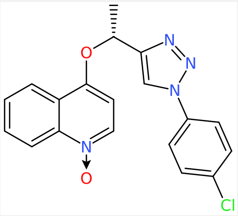	CHEMBL549612	0.0092			5.00nM	
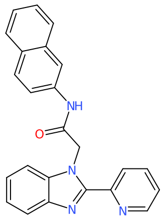	CHEMBL1957258	0.0092			13.5nM	

structure	chembl id	τ	IC50	EC50	Ki	Kd
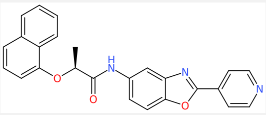	CHEMBL2348823	0.0092			28.8nM	
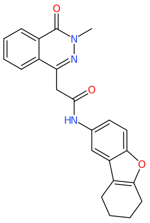	CHEMBL2315283	0.0077			15.7nM	
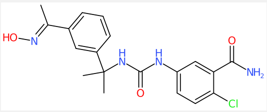	CHEMBL2178617	0.0067			3.46nM	
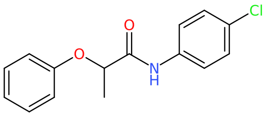	CHEMBL561640	0.012		3.30μM		

tid: 100264

· chembl id: CHEMBL612444

name: Mitochondrial complex V; ATP synthase:Bos taurus

similarity analysis

structure	chembl id	τ	IC50	EC50	Ki	Kd
global result		0.034: IC50	704nM			
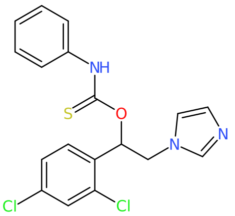	CHEMBL8244	0.018	3.60µM			
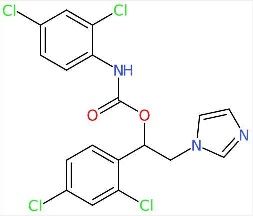	CHEMBL416142	0.018	6.77µM			
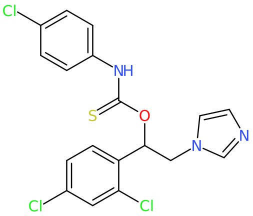	CHEMBL8376	0.018	430nM			
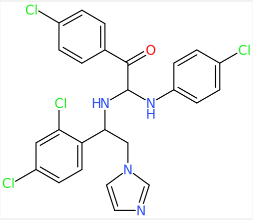	CHEMBL8024	0.012	82.0nM			
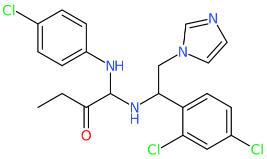	CHEMBL7894	0.012	2.27µM			

tid:	10980
chembl id:	CHEMBL279
name:	Vascular endothelial growth factor receptor 2:Homo sapiens

similarity analysis

structure	chembl id	τ	IC50	EC50	Ki	Kd
global result		0.033: IC50	151nM	65.2nM	95.6nM	123nM
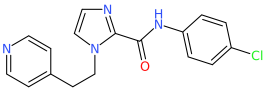	CHEMBL234202	0.018	708nM			
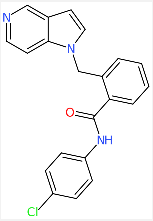	CHEMBL203003	0.018	92.0nM			
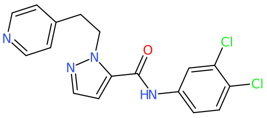	CHEMBL392330	0.012	177nM			
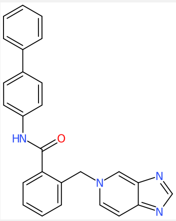	CHEMBL382478	0.012	690nM			
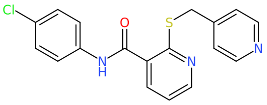	CHEMBL256556	0.012	26.0nM			
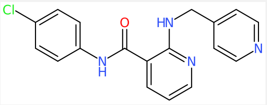	CHEMBL250228	0.012			8.00nM	
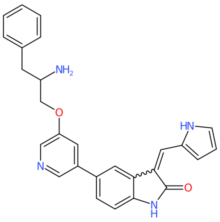	CHEMBL1980407	0.012			0.63nM	
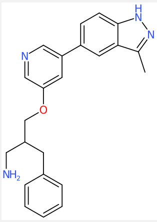	CHEMBL2000271	0.012			5.01µM	
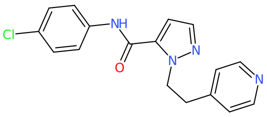	CHEMBL397323	0.012			99.5nM	

structure	chembl id	τ	IC50	EC50	Ki	Kd
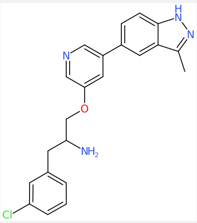	CHEMBL1974870	0.012			3.98μM	
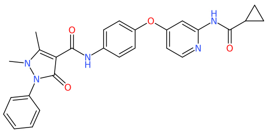	CHEMBL3703197	0.0077				2.90nM
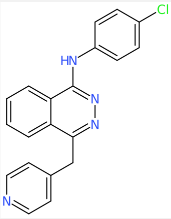	CHEMBL101253	0.0077				64.6nM
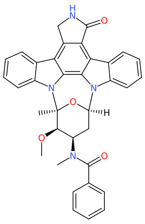	CHEMBL608533	0.0077				3.20μM
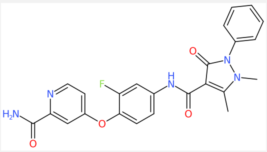	CHEMBL3703195	0.0077				450nM
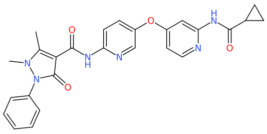	CHEMBL3703194	0.0077				3.20nM
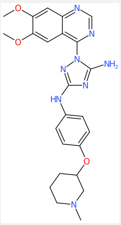	CHEMBL4098219	0.0077		4.00nM		
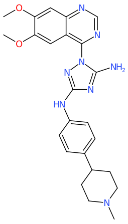	CHEMBL4090507	0.0077		14.0nM		
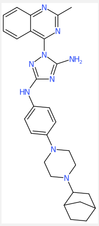	CHEMBL4083641	0.0067		104nM		

structure	chembl id	τ	IC50	EC50	Ki	Kd
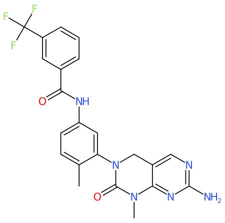	CHEMBL459850	0.0067		22.0nM		
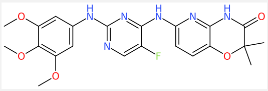	CHEMBL475251	0.0067		36.0nM		

tid:	104749
chembl id:	CHEMBL2095164
name:	Geranylgeranyl transferase type I:Homo sapiens

similarity analysis

structure	chembl id	τ	IC50	EC50	Ki	Kd
global result		0.033: IC50	883nM	389nM	8.00nM	
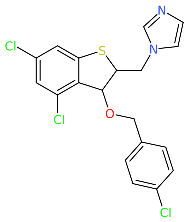	CHEMBL133288	0.018	18.8µM			
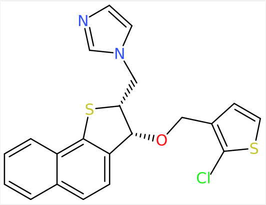	CHEMBL133625	0.018	15.0µM			
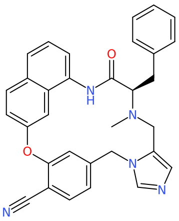	CHEMBL53862	0.012	350nM			
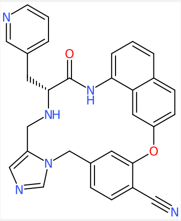	CHEMBL52859	0.012	147nM			
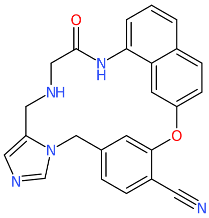	CHEMBL52975	0.012	675nM			
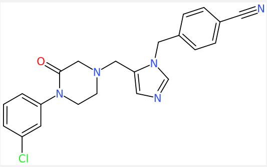	CHEMBL279433	0.0077		6.80µM		
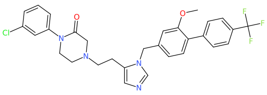	CHEMBL23460	0.0067		3.40µM		

structure	chembl id	τ	IC50	EC50	Ki	Kd
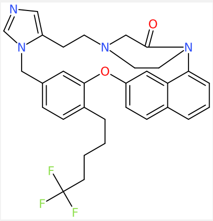	CHEMBL23650	0.0067		800nM		
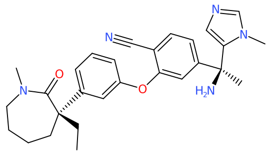	CHEMBL294888	0.0067		470nM		
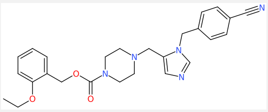	CHEMBL63107	0.0067		71.0nM		
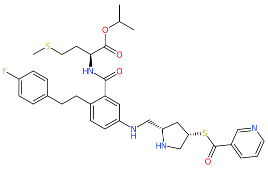	CHEMBL53670	0.0067			8.00nM	

tid:	10979
chembl id:	CHEMBL1868
name:	Vascular endothelial growth factor receptor 1:Homo sapiens

similarity analysis

structure	chembl id	τ	IC50	EC50	Ki	Kd
global result		0.033: IC50	255nM	454nM	59.8nM	116nM
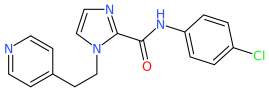	CHEMBL234202	0.018	940nM			
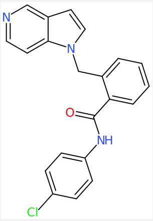	CHEMBL203003	0.018	220nM			
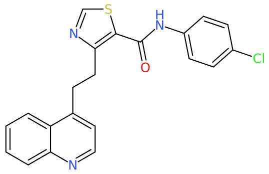	CHEMBL203149	0.012	6.85µM			
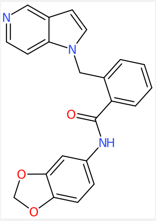	CHEMBL425587	0.012	180nM			
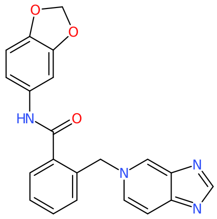	CHEMBL203167	0.012	360nM			
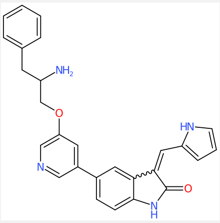	CHEMBL1980407	0.012			0.63nM	
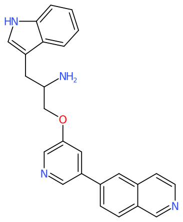	CHEMBL1981133	0.0092			398nM	

structure	chembl id	τ	IC50	EC50	Ki	Kd
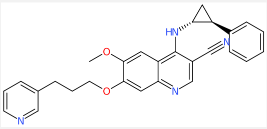	CHEMBL248391	0.0092			1.90µM	
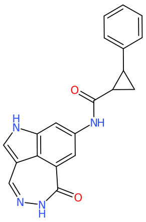	CHEMBL1969502	0.0092			39.8nM	
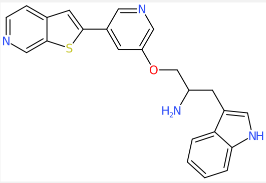	CHEMBL1966524	0.0092			316nM	
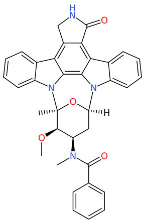	CHEMBL608533	0.0077				450nM
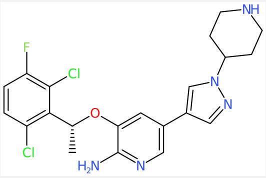	CHEMBL601719	0.0077				2.30µM
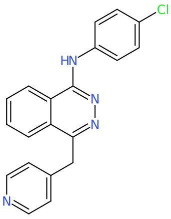	CHEMBL101253	0.0077				9.60nM
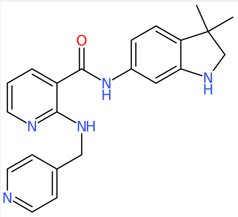	CHEMBL572881	0.0077				12.0nM
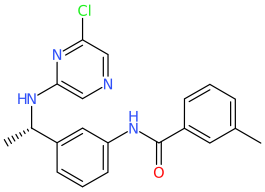	CHEMBL504075	0.0077				5.70nM
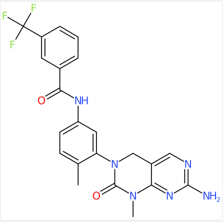	CHEMBL459850	0.0067		236nM		

structure	chembl id	τ	IC50	EC50	Ki	Kd
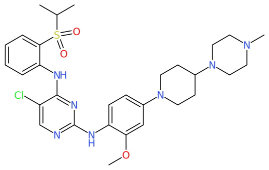	CHEMBL509032	0.0067		872nM		

tid:	11300
chembl id:	CHEMBL4124
name:	Histamine H3 receptor:Rattus norvegicus

similarity analysis

structure	chembl id	τ	IC50	EC50	Ki	Kd
global result		0.033: Ki	15.3nM	3.97nM	14.9nM	6.42nM
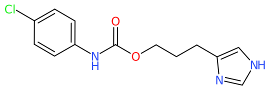	CHEMBL16231	0.018			4.09nM	
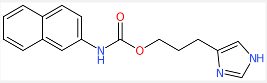	CHEMBL16274	0.018			120nM	
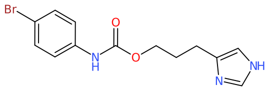	CHEMBL277989	0.012			18.9nM	
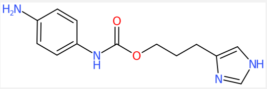	CHEMBL15842	0.012			355nM	
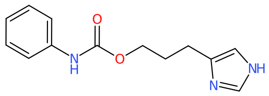	CHEMBL16142	0.012			13.3nM	
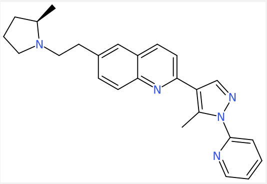	CHEMBL393581	0.0077		3.63nM		
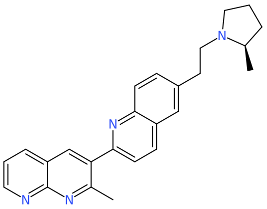	CHEMBL263101	0.0077		7.24nM		
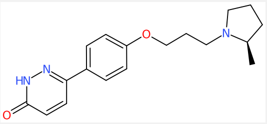	CHEMBL1829335	0.0067		2.00nM		
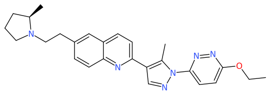	CHEMBL237394	0.0067		0.74nM		
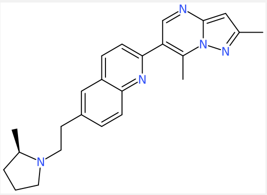	CHEMBL392573	0.0067		5.01nM		
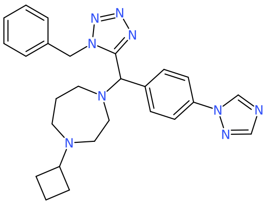	CHEMBL1223235	0.0077	9.20nM			
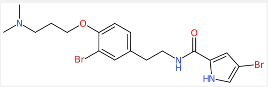	CHEMBL493091	0.0067	2.35μM			

structure	chembl id	τ	IC50	EC50	Ki	Kd
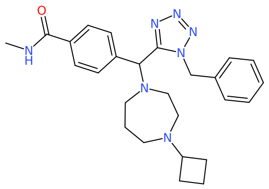	CHEMBL1221429	0.0067	12.0nM			
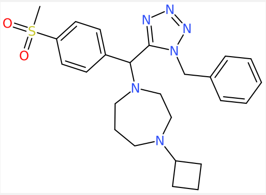	CHEMBL1223157	0.0067	17.0nM			
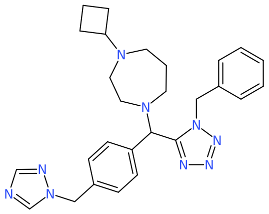	CHEMBL1223236	0.0067	17.0nM			
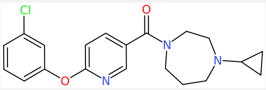	CHEMBL1171001	0.0067				11.5nM
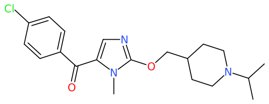	CHEMBL481745	0.0067				10.0nM
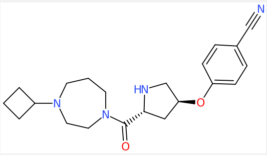	CHEMBL1097881	0.006				12.6nM
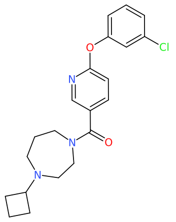	CHEMBL1171002	0.006				5.01nM
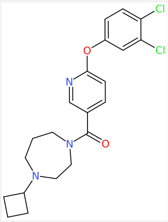	CHEMBL1171936	0.006				6.92nM

tid: 10635

chembl id: CHEMBL2492

name: Neuronal acetylcholine receptor protein alpha-7 subunit:Homo sapiens

similarity analysis

structure	chembl id	τ	IC50	EC50	Ki	Kd
global result		0.033: Ki	2.30µM	705nM	207nM	19.3nM
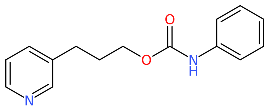	CHEMBL1255822	0.018			14.5µM	
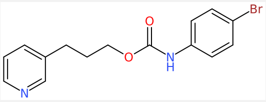	CHEMBL1255823	0.018			6.92µM	
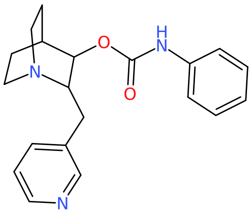	CHEMBL3640294	0.012			7.00nM	
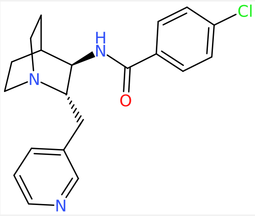	CHEMBL2179842	0.012			15.0nM	
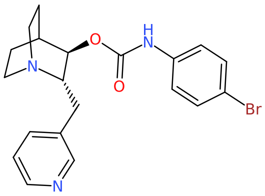	CHEMBL1258240	0.012			2.69nM	
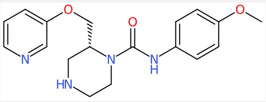	CHEMBL3303977	0.018	19.0nM			
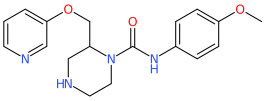	CHEMBL3302229	0.018	11.0nM			
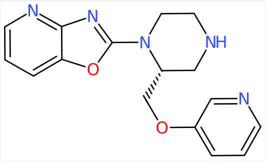	CHEMBL3302240	0.0092	6.00nM			
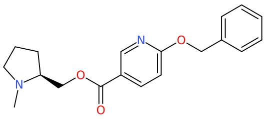	CHEMBL2381567	0.0077	59.1µM			
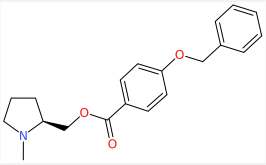	CHEMBL2381566	0.0077	2.30µM			

structure	chembl id	τ	IC50	EC50	Ki	Kd
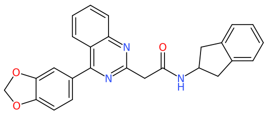	CHEMBL1210643	0.0092		25.1nM		
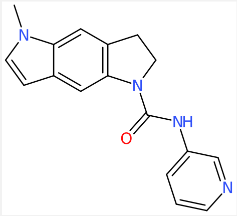	CHEMBL297784	0.0092		1.50µM		
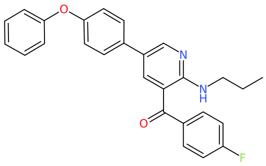	CHEMBL2440317	0.0092		560nM		
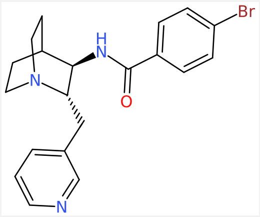	CHEMBL1258005	0.0092		50.0nM		
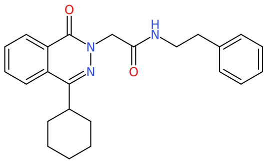	CHEMBL1210579	0.0092		10.0nM		
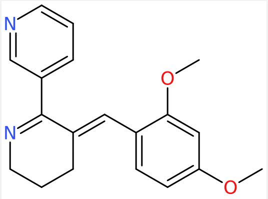	CHEMBL134713	0.0077				158nM
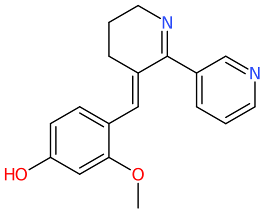	CHEMBL1162395	0.0067				23.0nM
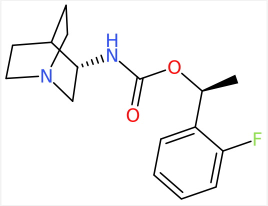	CHEMBL499536	0.006				200nM
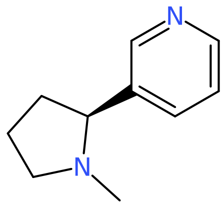	CHEMBL3	0.005				1.06nM
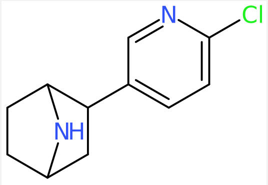	CHEMBL6623	0.005				2.59nM

tid:	17074
chembl id:	CHEMBL3943
name:	Histamine H1 receptor:Cavia porcellus

similarity analysis

structure	chembl id	τ	IC50	EC50	Ki	Kd
global result		0.033: Ki	176nM	111nM	334nM	242nM
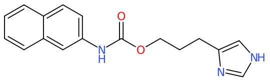	CHEMBL16274	0.018			7.94µM	
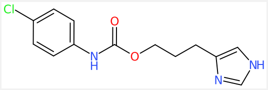	CHEMBL16231	0.018			63.1µM	
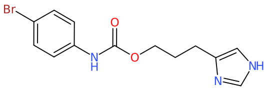	CHEMBL277989	0.012			25.1µM	
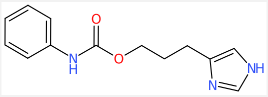	CHEMBL16142	0.012			10.00µM	
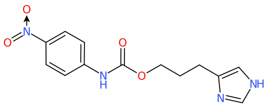	CHEMBL15883	0.012			20.0µM	
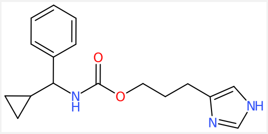	CHEMBL128585	0.0092				31.6µM
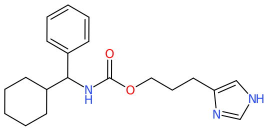	CHEMBL131476	0.0092				7.94µM
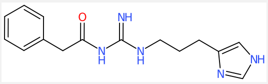	CHEMBL499092	0.0077				575nM
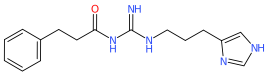	CHEMBL470790	0.0077				2.14µM
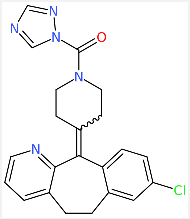	CHEMBL3401455	0.0077				155nM
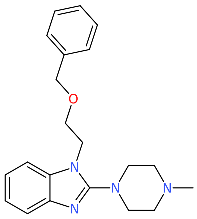	CHEMBL17808	0.0092	430nM			
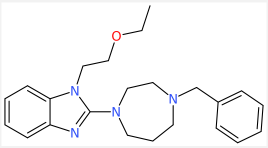	CHEMBL18255	0.0077	15.0nM			

structure	chembl id	τ	IC50	EC50	Ki	Kd
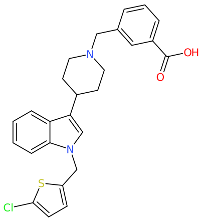	CHEMBL185489	0.0077	184nM			
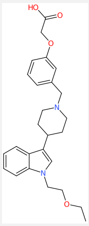	CHEMBL373534	0.0077	130nM			
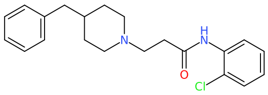	CHEMBL100276	0.0077	561nM			
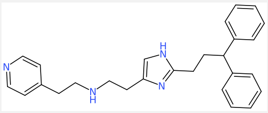	CHEMBL424336	0.0067		115nM		
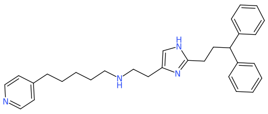	CHEMBL436415	0.0067		95.5nM		
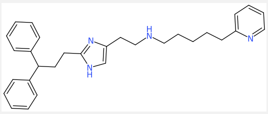	CHEMBL153468	0.0067		15.5nM		
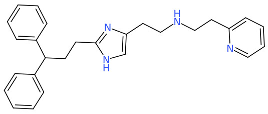	CHEMBL153067	0.0067		41.7nM		
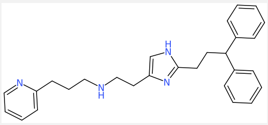	CHEMBL153321	0.0067		21.4nM		

tid:	10116
chembl id:	CHEMBL2882
name:	Histamine H2 receptor:Cavia porcellus

similarity analysis

structure	chembl id	τ	IC50	EC50	Ki	Kd
global result		0.033: Ki	2.15µM	80.5nM	3.77µM	1.16µM
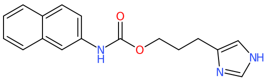	CHEMBL16274	0.018			7.94µM	
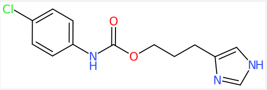	CHEMBL16231	0.018			79.4µM	
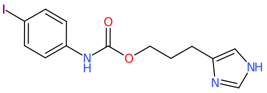	CHEMBL16874	0.012			5.01µM	
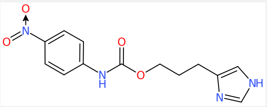	CHEMBL15883	0.012			12.6µM	
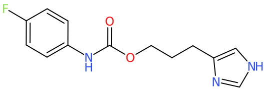	CHEMBL274989	0.012			63.1µM	
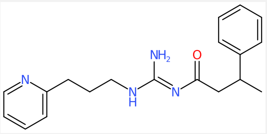	CHEMBL3220665	0.0092		195nM		
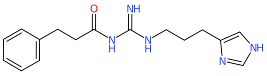	CHEMBL470790	0.0077		166nM		
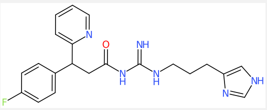	CHEMBL472273	0.0077		33.9nM		
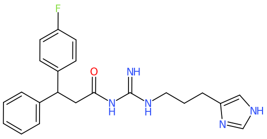	CHEMBL469749	0.0077		54.9nM		
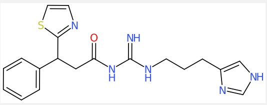	CHEMBL472441	0.0077		23.1nM		
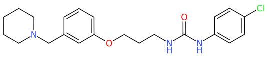	CHEMBL81520	0.0077				631nM
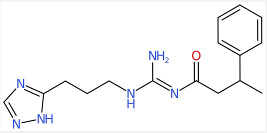	CHEMBL3220635	0.0077				479nM
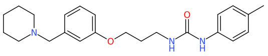	CHEMBL311272	0.0077				631nM
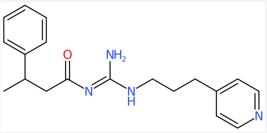	CHEMBL3220886	0.0077				19.1µM
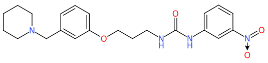	CHEMBL311978	0.0077				269nM

structure	chembl id	τ	IC50	EC50	Ki	Kd
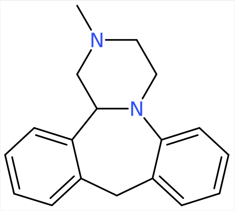	CHEMBL6437	0.006	880nM			
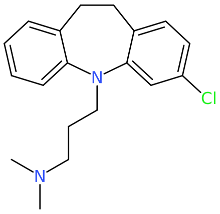	CHEMBL415	0.006	720nM			
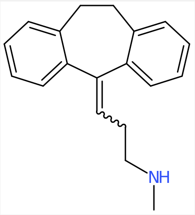	CHEMBL445	0.006	7.00µM			
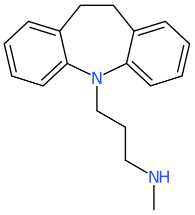	CHEMBL72	0.006	3.80µM			
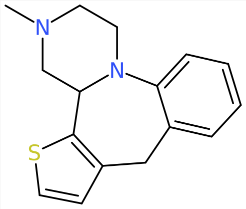	CHEMBL13645	0.006	19.0µM			

tid:	101435
chembl id:	CHEMBL6084
name:	Acetylcholine-binding protein:Lymnaea stagnalis

similarity analysis

structure	chembl id	τ	IC50	EC50	Ki	Kd
global result		0.032: Kd			663nM	670nM
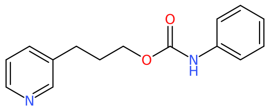	CHEMBL1255822	0.018			282µM	741µM
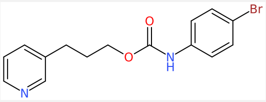	CHEMBL1255823	0.018				50.1µM
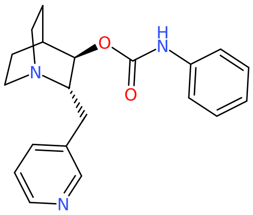	CHEMBL1258126	0.012			61.7nM	100.0nM
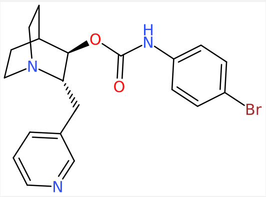	CHEMBL1258240	0.012			18.2nM	24.0nM
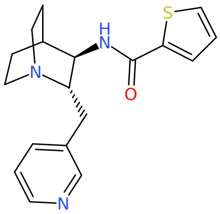	CHEMBL1258125	0.0092			148nM	240nM
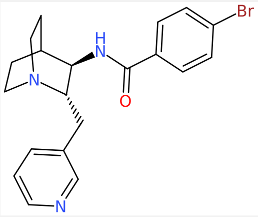	CHEMBL1258005	0.0092			89.1nM	

tid:	14042
chembl id:	CHEMBL4011
name:	Human immunodeficiency virus type 1 Tat protein:Human immunodeficiency virus type 1 group M subtype B (isolate HXB2)(HIV-1)

similarity analysis

structure	chembl id	τ	IC50	EC50	Ki	Kd
global result		0.031: EC50	10.6µM	2.24µM		3.72µM
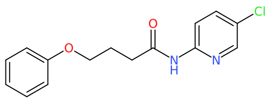	CHEMBL1386792	0.024		6.70µM		
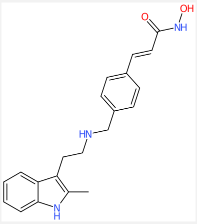	CHEMBL483254	0.0077		130nM		
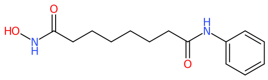	CHEMBL98	0.0067		2.30µM		
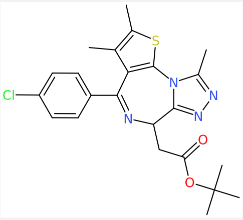	CHEMBL2132936	0.0067		8.10µM		
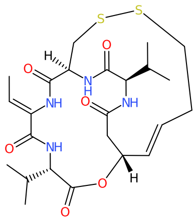	CHEMBL3310505	0.0067		220nM		
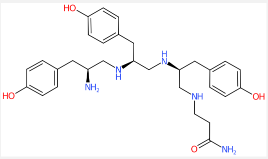	CHEMBL1081563	0.006	19.0µM			
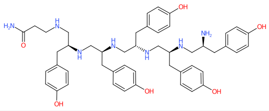	CHEMBL1075829	0.005	30.0µM			
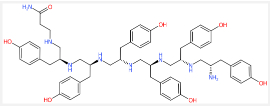	CHEMBL1075830	0.005	7.00µM			
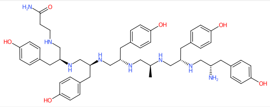	CHEMBL1075815	0.005	6.00µM			
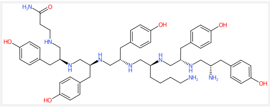	CHEMBL1075821	0.005	12.0µM			

structure	chembl id	τ	IC50	EC50	Ki	Kd
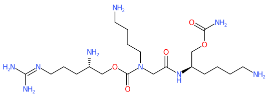	CHEMBL196072	0.005				1.80µM
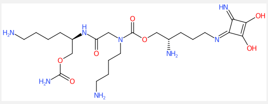	CHEMBL371627	0.005				7.70µM

tid: 194

chembl id: CHEMBL244

name: Coagulation factor X:Homo sapiens

similarity analysis

structure	chembl id	τ	IC50	EC50	Ki	Kd
global result		0.03: Ki	77.6nM		70.9nM	33.2nM
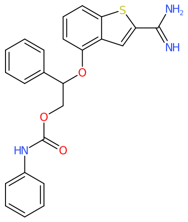	CHEMBL598400	0.018			1.81µM	
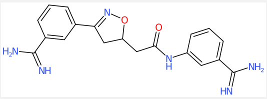	CHEMBL94563	0.012			1.80µM	
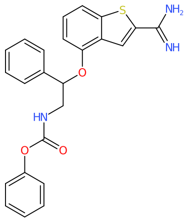	CHEMBL597370	0.012			560nM	
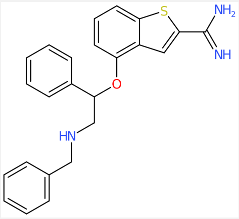	CHEMBL599001	0.012			250nM	
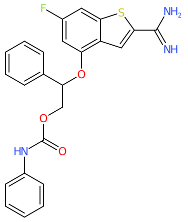	CHEMBL602083	0.012			1.59µM	
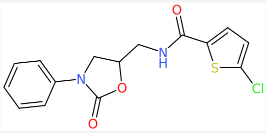	CHEMBL3671231	0.012	2.00µM			
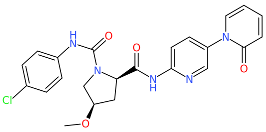	CHEMBL476187	0.012	4.60nM			
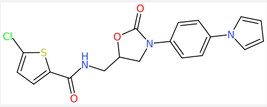	CHEMBL3671221	0.012	1.70µM			

structure	chembl id	τ	IC50	EC50	Ki	Kd
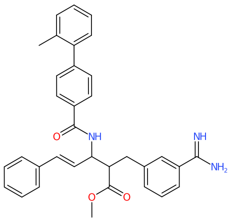	CHEMBL138299	0.0092	90.0nM			
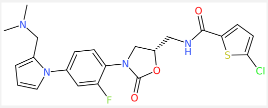	CHEMBL3415983	0.0092	179nM			
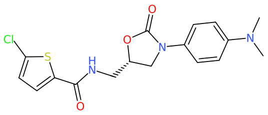	CHEMBL198654	0.0092				14.7nM
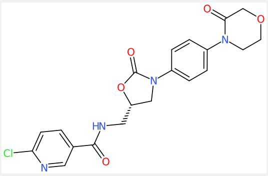	CHEMBL436293	0.0092				99.2nM
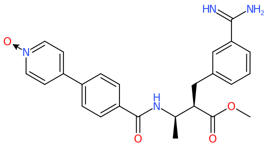	CHEMBL46618	0.0092				38.8nM
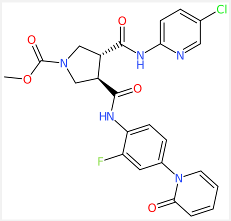	CHEMBL1221916	0.0092				4.30nM
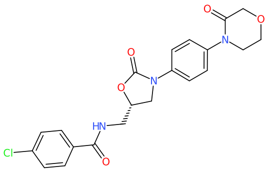	CHEMBL196492	0.0092				17.7nM

tid:	11442
chembl id:	CHEMBL2568
name:	Liver glycogen phosphorylase:Homo sapiens

similarity analysis

structure	chembl id	τ	IC50	EC50	Ki	Kd
global result		0.03: IC50	296nM		3.35µM	2.12µM
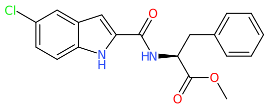	CHEMBL100872	0.018	140nM			
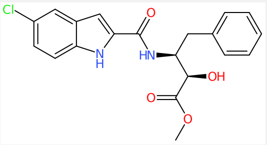	CHEMBL319854	0.012	210nM			
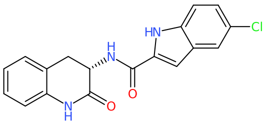	CHEMBL134854	0.012	47.0nM			
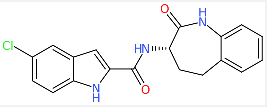	CHEMBL136712	0.012	240nM			
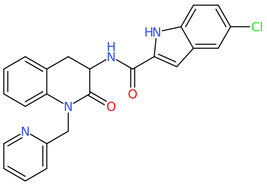	CHEMBL136812	0.012	110nM			
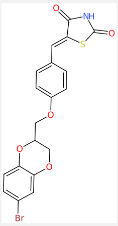	CHEMBL243412	0.0092			80.0µM	
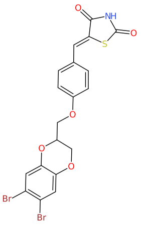	CHEMBL242918	0.0077			12.0µM	
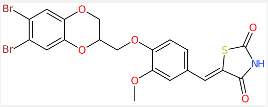	CHEMBL245060	0.0077			30.0µM	
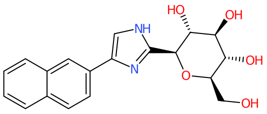	CHEMBL3770455	0.006			26.0nM	
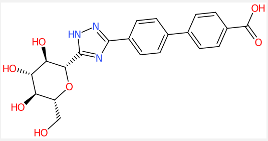	CHEMBL417267[illegible]	0.006			2.67µM	

structure	chembl id	τ	IC50	EC50	Ki	Kd
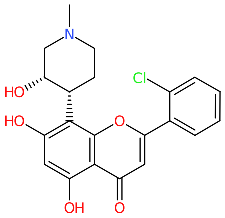	CHEMBL428690	0.006				2.12µM

tid:	193
chembl id:	CHEMBL2016
name:	Coagulation factor IX:Homo sapiens

similarity analysis

structure	chembl id	τ	IC50	EC50	Ki	Kd
global result		0.03: Ki	203nM		34.9nM	
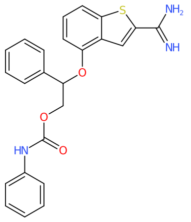	CHEMBL598400	0.018			10.0nM	
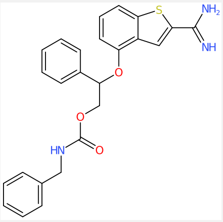	CHEMBL605842	0.012			61.0nM	
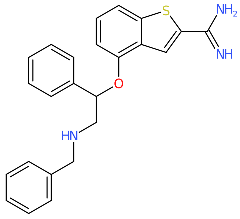	CHEMBL599001	0.012			100.0nM	
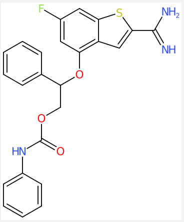	CHEMBL602083	0.012			15.5nM	
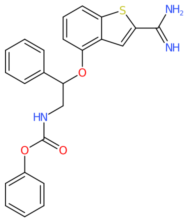	CHEMBL597370	0.012			50.0nM	
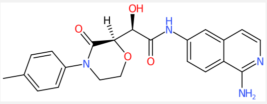	CHEMBL3658386	0.0092	20.0nM			
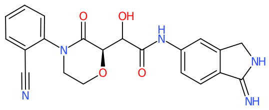	CHEMBL3658394	0.0092	66.0nM			
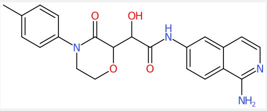	CHEMBL3658388	0.0092	40.0nM			
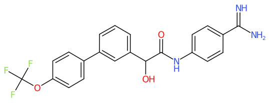	CHEMBL3924571	0.0077	445nM			

structure	chembl id	τ	IC50	EC50	Ki	Kd
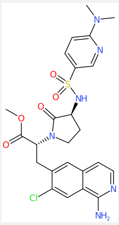	CHEMBL3929161	0.0077	5.60µM			

tid:	11942
chembl id:	CHEMBL3286
name:	Urokinase-type plasminogen activator:Homo sapiens

similarity analysis

structure	chembl id	τ	IC50	EC50	Ki	Kd
global result		0.03: Ki	1.62µM		977nM	
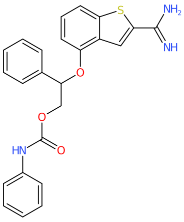	CHEMBL598400	0.018			440nM	
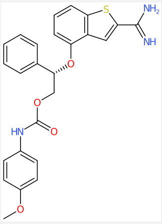	CHEMBL604294	0.012			1.63µM	
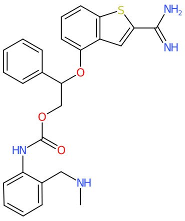	CHEMBL598989	0.012			308nM	
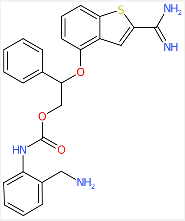	CHEMBL599394	0.012			297nM	
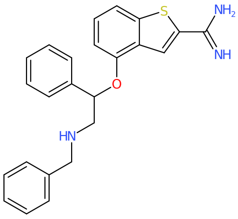	CHEMBL599001	0.012			310nM	
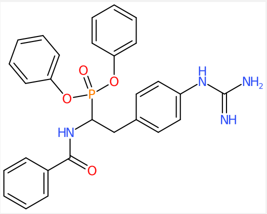	CHEMBL393592	0.012	114nM			
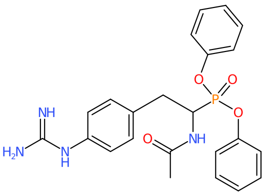	CHEMBL239747	0.012	7.20nM			

structure	chembl id	τ	IC50	EC50	Ki	Kd
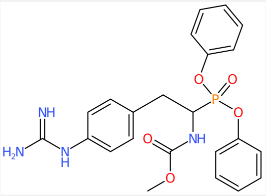	CHEMBL393591	0.012	3.10nM			
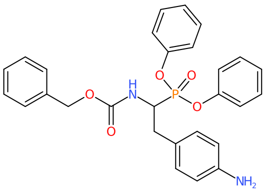	CHEMBL3771195	0.012	19.8µM			
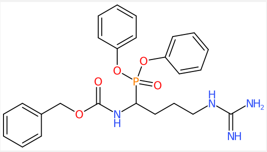	CHEMBL239331	0.012	840nM			

tid:	13077
chembl id:	CHEMBL338
name:	Dopamine transporter:Rattus norvegicus

similarity analysis

structure	chembl id	τ	IC50	EC50	Ki	Kd
global result		0.03: Ki	116nM	771nM	150nM	4.65nM
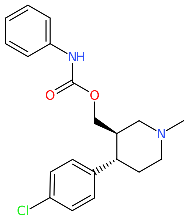	CHEMBL103805	0.018			91.0nM	
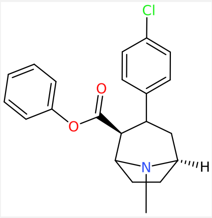	CHEMBL93721	0.012	1.98nM		5.25nM	
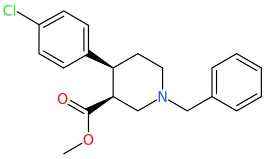	CHEMBL211248[illegible]	0.012			3.28μM	
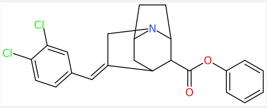	CHEMBL59545	0.012			4.44μM	
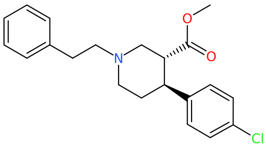	CHEMBL2112481	0.012			1.08μM	
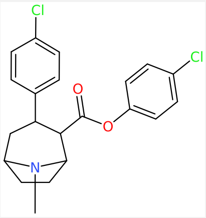	CHEMBL420343	0.012	55.0nM			
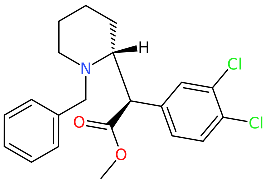	CHEMBL1254674	0.012	76.1nM			
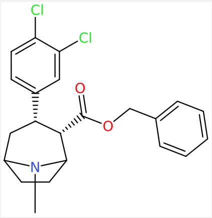	CHEMBL328204	0.012	19.0nM			

structure	chembl id	τ	IC50	EC50	Ki	Kd
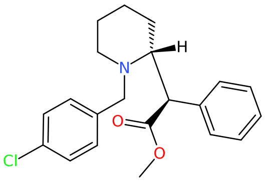	CHEMBL1253840	0.012	31.0nM			
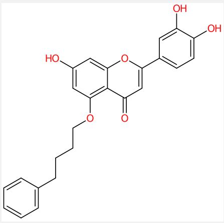	CHEMBL1278046	0.0077		2.59μM		
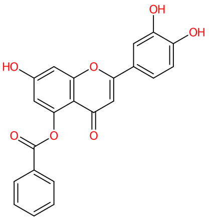	CHEMBL1277609	0.0067		11.2μM		
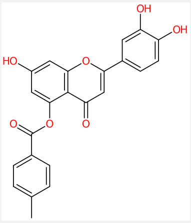	CHEMBL1277610	0.0067		6.92μM		
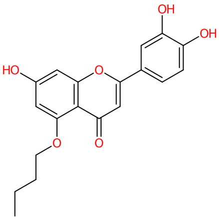	CHEMBL1277795	0.006		2.40μM		
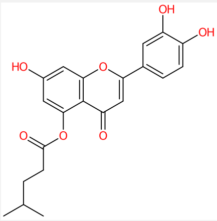	CHEMBL1277515	0.006		2.71μM		
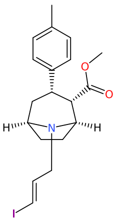	CHEMBL538876	0.0077				4.00nM
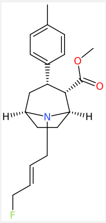	CHEMBL1947086	0.0077				9.00nM
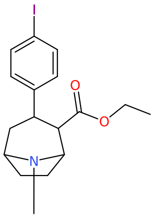	CHEMBL23714	0.0067				1.00nM

structure	chembl id	τ	IC50	EC50	Ki	Kd
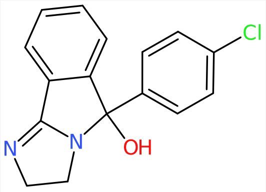 	CHEMBL781	0.005				8.63nM
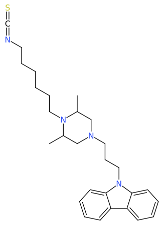	CHEMBL138438	0.005				8.91nM

tid:	11289
chembl id:	CHEMBL3229
name:	Anandamide amidohydrolase:Rattus norvegicus

similarity analysis

structure	chembl id	τ	IC50	EC50	Ki	Kd
global result		0.03: IC50	246nM	212nM	36.0nM	0.60nM
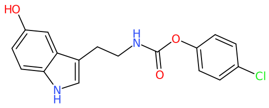	CHEMBL241137	0.018	6.80µM			
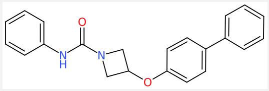	CHEMBL3677685	0.012	135nM			
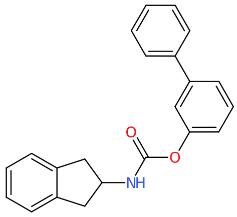	CHEMBL485886	0.012	214nM			
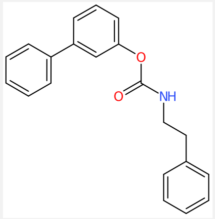	CHEMBL520360	0.012	479nM			
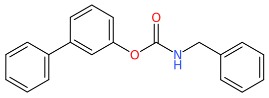	CHEMBL485689	0.012	138nM			
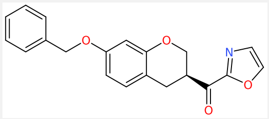	CHEMBL3264469	0.0092			16.0nM	
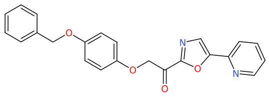	CHEMBL387680	0.0092			5.30µM	
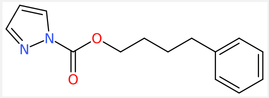	CHEMBL3318621	0.0092			710nM	
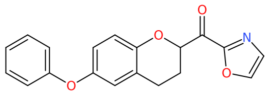	CHEMBL3264460	0.0092			23.0nM	
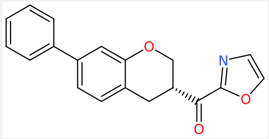	CHEMBL3264464	0.0092			20.0nM	
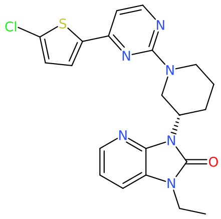	CHEMBL1761301	0.0092		280nM		

structure	chembl id	τ	IC50	EC50	Ki	Kd
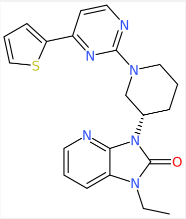	CHEMBL176130[illegible]	0.0077		1.80µM		
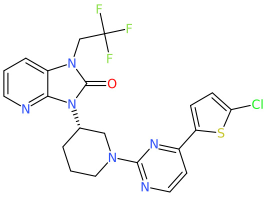	CHEMBL1761302	0.0077		100.0nM		
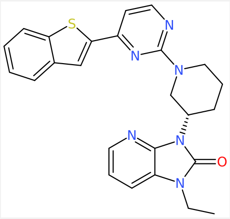	CHEMBL1761303	0.0077		80.0nM		
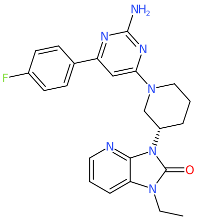	CHEMBL1761304	0.0077		100.0nM		
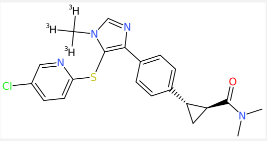	CHEMBL2386571	0.0067				0.60nM

tid:	20059
· chembl id:	CHEMBL5035
name:	Heme oxygenase 1:Rattus norvegicus

similarity analysis

structure	chembl id	τ	IC50	EC50	Ki	Kd
global result		0.03: IC50	4.03µM			
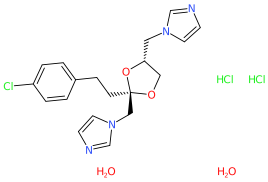	CHEMBL459907	0.018	10.00µM			
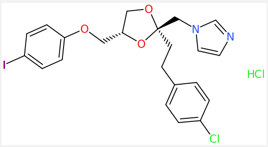	CHEMBL474961	0.012	9.00µM			
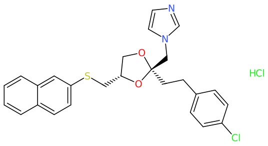	CHEMBL472975	0.012	900nM			
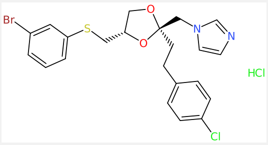	CHEMBL472782	0.012	5.00µM			
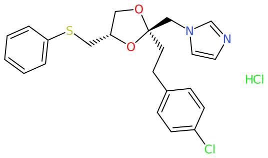	CHEMBL475163	0.012	1.03µM			

tid: 228

chembl id: CHEMBL247

name: Human immunodeficiency virus type 1 reverse transcriptase:Human immunodeficiency virus 1

similarity analysis

structure	chembl id	τ	IC50	EC50	Ki	Kd
global result		0.03: IC50	653nM	339nM	212nM	10.5µM
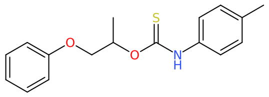	CHEMBL471691	0.018	1.40µM			
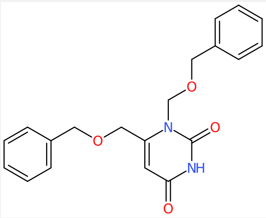	CHEMBL3797342	0.012	9.07µM			
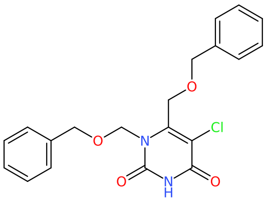	CHEMBL3800107	0.012	9.70µM			
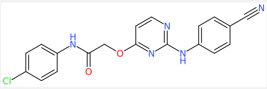	CHEMBL3397636	0.012	26.9µM			
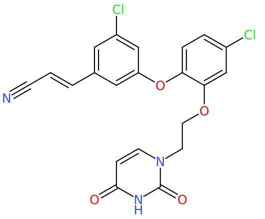	CHEMBL1923490	0.012	3.00nM			
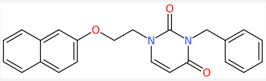	CHEMBL2337189	0.012			1.00µM	
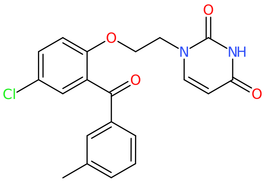	CHEMBL1835505	0.012			210nM	
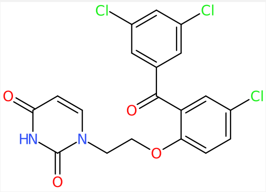	CHEMBL1835510	0.012			2.83µM	
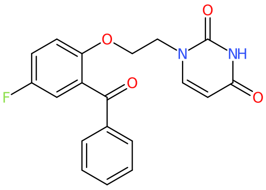	CHEMBL1835502	0.012			2.60µM	

structure	chembl id	τ	IC50	EC50	Ki	Kd
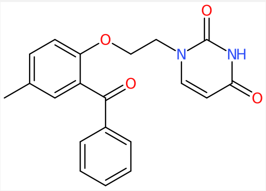	CHEMBL1835501	0.012			1.70µM	
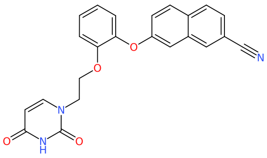	CHEMBL3983638	0.012		3.22µM		
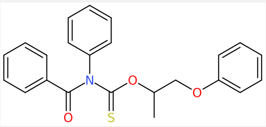	CHEMBL350574	0.012		1.30µM		
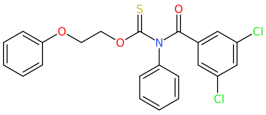	CHEMBL165232	0.012		8.80µM		
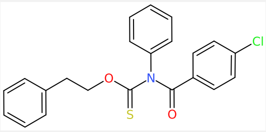	CHEMBL164496	0.012		4.00µM		
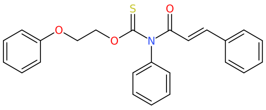	CHEMBL165121	0.012		7.70µM		
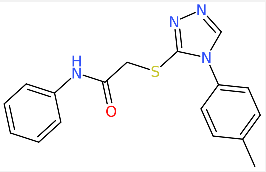	CHEMBL1671953	0.012				800µM
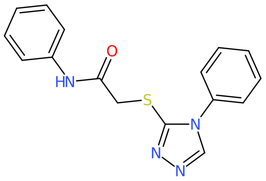	CHEMBL1468531	0.012				1.70mM
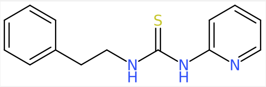	CHEMBL316796	0.0077				6.00µM
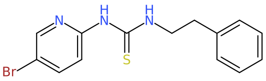	CHEMBL317356	0.0077				2.00µM
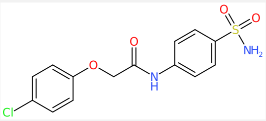	CHEMBL1535399	0.0077				30.0µM

tid:	261
· chembl id:	CHEMBL1811
name:	Prostanoid EP1 receptor:Homo sapiens

similarity analysis

structure	chembl id	τ	IC50	EC50	Ki	Kd
global result		0.03: IC50	36.7nM	277nM	291nM	60.4nM
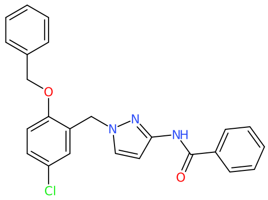	CHEMBL255485	0.018	251nM			
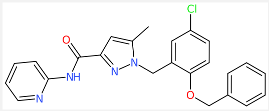	CHEMBL257147	0.012	126nM			
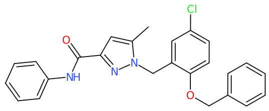	CHEMBL404698	0.012	39.8nM			
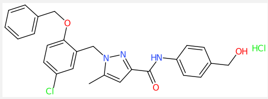	CHEMBL520438	0.012	6.31nM		39.8nM	
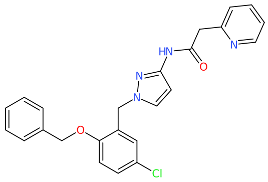	CHEMBL402326	0.012	398nM			
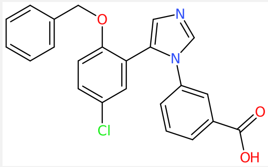	CHEMBL207293	0.012			3.55nM	
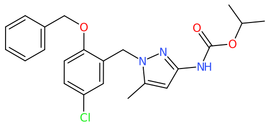	CHEMBL256092	0.012			50.1nM	7.94nM
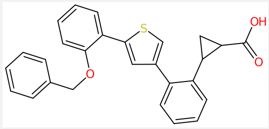	CHEMBL125588	0.0092			48.0μM	
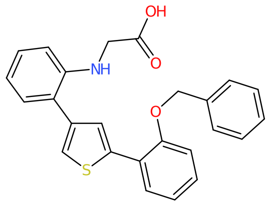	CHEMBL124675	0.0092			85.0μM	

structure	chembl id	τ	IC50	EC50	Ki	Kd
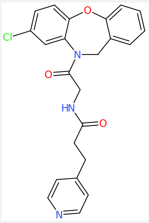	CHEMBL347397	0.0092				2.00µM
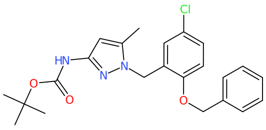	CHEMBL258199	0.0092				50.1nM
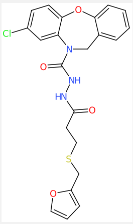	CHEMBL358653	0.0077				7.94nM
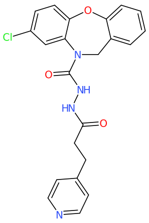	CHEMBL155358	0.0077				316nM
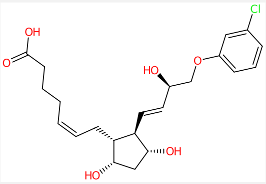	CHEMBL37853	0.0067		760nM		
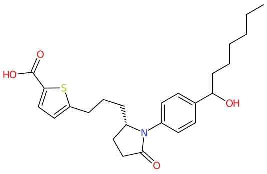	CHEMBL3947001	0.0067		6.95µM		
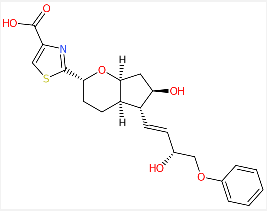	CHEMBL3804978	0.0067		1.40nM		
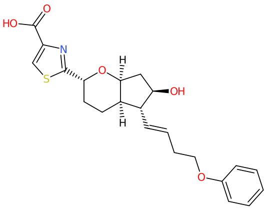	CHEMBL3805176	0.0067		160nM		
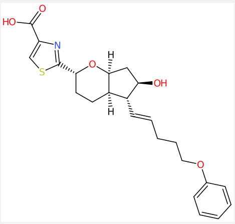	CHEMBL3805169	0.0067		970nM		
