# Supplementary material for: PLATO: A Predictive Drug Discovery Web Platform for Efficient Target Fishing and Bioactivity Profiling of Small Molecules
Source: Int J Mol Sci. 2022 May 8;23(9):5245. doi: 10.3390/ijms23095245 (PMC9103655; doi:10.3390/ijms23095245)
Supplement: Supplementary file 1 [file ijms-23-05245-s001.zip › Target_Fishing_Case_study_2.pdf]

target fishing

smiles: O=C(Nc1ccc(Cl)cc1)OC(COc2ccccc2)Cn3ccnc3

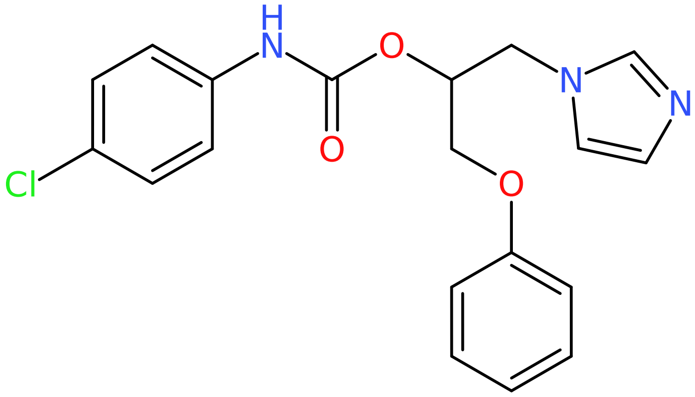

Target	score	reliable
Cytochrome P450 19A1:Homo sapiens	4.22	no
Mitochondrial complex V; ATP synthase:Bos taurus	3.19	no
Thromboxane-A synthase:Homo sapiens	2.96	no
Cytochrome P450 1A2:Homo sapiens	2.60	no
Cytochrome P450 2C19:Homo sapiens	2.60	no
Cytochrome P450 2C9:Homo sapiens	2.60	no
Beta-1 adrenergic receptor:Homo sapiens	2.30	no
Cytochrome P450 2D6:Homo sapiens	2.30	no
Heme oxygenase 1:Rattus norvegicus	2.26	no
Vascular endothelial growth factor receptor 2:Homo sapiens	1.83	no
Beta-2 adrenergic receptor:Canis lupus familiaris	1.75	no
15-cis-phytoene desaturase:Synechococcus elongatus (strain PCC 7942) (Anacystis nidulans R2)	1.70	no
Prostanoid EP1 receptor:Homo sapiens	1.47	no
Cytochrome P450 3A4:Homo sapiens	1.42	no
Beta-3 adrenergic receptor:Homo sapiens	1.38	no
Neuronal acetylcholine receptor protein alpha-7 subunit:Rattus norvegicus	1.21	no
Metabotropic glutamate receptor 5:Homo sapiens	1.20	no
Histamine H2 receptor:Cavia porcellus	1.10	no
Histamine H3 receptor:Rattus norvegicus	1.10	no
Histamine H1 receptor:Cavia porcellus	1.10	no
Sterol 14-alpha demethylase:Trypanosoma cruzi (strain CL Brener)	1.06	no
Vascular endothelial growth factor receptor 1:Homo sapiens	1.00	no
Anandamide amidohydrolase:Rattus norvegicus	0.93	no
1-acylglycerol-3-phosphate O-acyltransferase beta:Homo sapiens	0.87	no
M18 aspartyl aminopeptidase:Plasmodium falciparum 3D7	0.87	no
Galanin receptor 3:Homo sapiens	0.87	no
Lysophosphatidic acid receptor Edg-2:Homo sapiens	0.87	no
P2X purinoceptor 7:Homo sapiens	0.86	no
P2X purinoceptor 7:Rattus norvegicus	0.86	no
Lysine-specific histone demethylase 1:Homo sapiens	0.86	no

tid: 65
· chembl id: CHEMBL1978
name: Cytochrome P450 19A1:Homo sapiens

similarity analysis

structure	mfp1	featmfp1	rdkit7	pattern	ap_bits	tt_bits	fp2	pubchem	cdk_maccs	graph	substructure	hybridization	klekota_roth
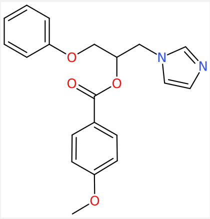 CHEMBL1485809 IC50:16.5nM	**0.66**		**0.55**		**0.53**	**0.61**	**0.54**			**0.64**		**0.62**	**0.52**
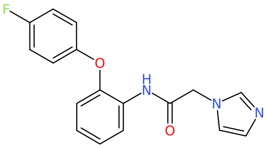 CHEMBL1784798 IC50:248nM		**0.72**						**0.84**	**0.73**				
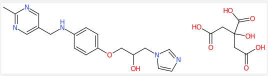 CHEMBL1784796 IC50:3.50µM				**0.87**									
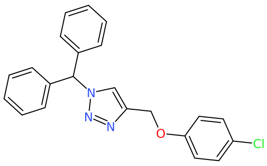 CHEMBL1824763 IC50:13.4µM											**0.79**		

tid: 100264

chembl id: CHEMBL612444

name: Mitochondrial complex V; ATP synthase:Bos taurus

similarity analysis

structure	mfp1	featmfp1	rdkit7	pattern	ap_bits	tt_bits	fp2	pubchem	cdk_maccs	graph	substructure	hybridization	klekota_roth
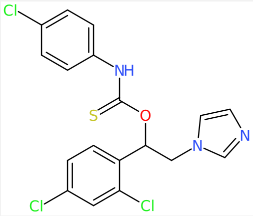 CHEMBL8376 IC50:430nM					**0.46**								
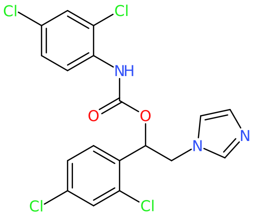 CHEMBL416142 IC50:6.77µM	**0.69**	**0.76**	**0.43**				**0.57**	**0.82**	**0.82**	**0.60**	**0.93**	**0.56**	**0.51**
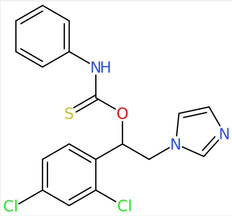 CHEMBL8244 IC50:3.60µM				**0.80**		**0.45**							

tid:	248
chembl id:	CHEMBL1835
name:	Thromboxane-A synthase:Homo sapiens

similarity analysis

structure	mfp1	featmfp1	rdkit7	pattern	ap_bits	tt_bits	fp2	pubchem	cdk_maccs	graph	substructure	hybridization	klekota_roth
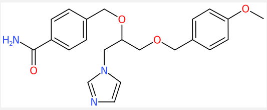 CHEMBL290637 IC50:6.00µM					0.47								
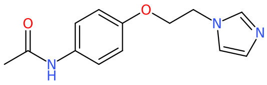 CHEMBL33690 IC50:4.00µM							0.54						
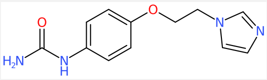 CHEMBL33151 IC50:840nM	0.57	0.68				0.48		0.86			0.80		
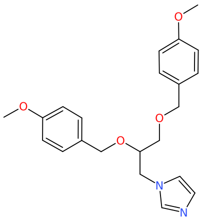 CHEMBL41441 IC50:9.90µM			0.46	0.85									
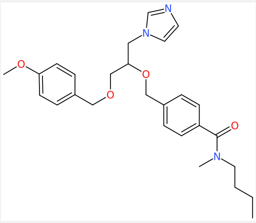 CHEMBL40983 IC50:5.40µM										0.62			
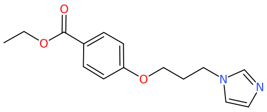 CHEMBL30604 IC50:3.00µM												0.50	

structure	mfp1	featmfp1	rdkit7	pattern	ap_bits	tt_bits	fp2	pubchem	cdk_maccs	graph	substructure	hybridization	klekota_roth
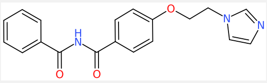 CHEMBL33874 IC50:180nM									**0.71**				
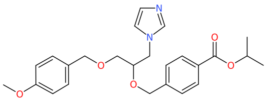 CHEMBL38988 IC50:1.61μM													**0.42**

tid: 12594
chembl id: CHEMBL3356
name: Cytochrome P450 1A2:Homo sapiens

similarity analysis

structure	mfp1	featmfp1	rdkit7	pattern	ap_bits	tt_bits	fp2	pubchem	cdk_maccs	graph	substructure	hybridization	klekota_roth
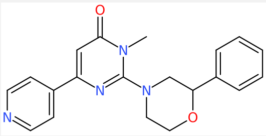 CHEMBL3091546 IC50:23.1µM					0.41								
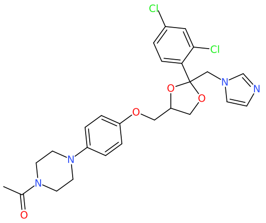 CHEMBL157101 IC50:26.0µM		0.63	0.36				0.46					0.37	
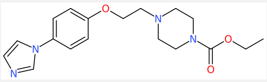 CHEMBL114555 IC50:2.24µM				0.89				0.84					
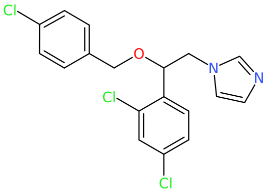 CHEMBL808 IC50:300nM						0.32							
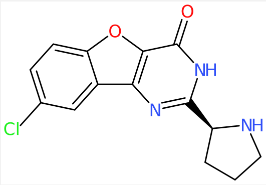 CHEMBL2030402 IC50:6.90µM										0.58			
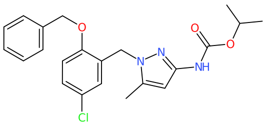 CHEMBL256092 IC50:7.80µM									0.73		0.88		0.53

structure	mfp1	featmfp1	rdkit7	pattern	ap_bits	tt_bits	fp2	pubchem	cdk_maccs	graph	substructure	hybridization	klekota_roth
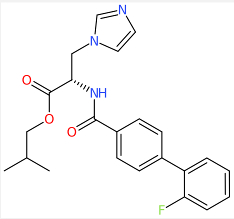 CHEMBL3984743 IC50:21.0μM	**0.47**												

tid:	12912
· chembl id:	CHEMBL3622
name:	Cytochrome P450 2C19:Homo sapiens

similarity analysis

structure	mfp1	featmfp1	rdkit7	pattern	ap_bits	tt_bits	fp2	pubchem	cdk_maccs	graph	substructure	hybridization	klekota_roth
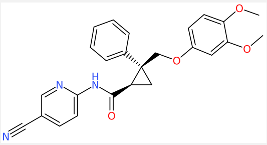 CHEMBL3343259 IC50:8.60µM					**0.41**								
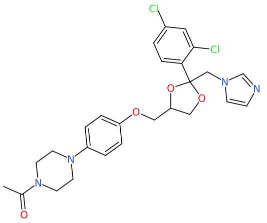 CHEMBL157101 IC50:15.4µM		**0.63**	**0.36**				**0.46**			**0.54**			
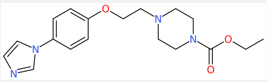 CHEMBL114555 IC50:469nM				**0.89**				**0.84**					
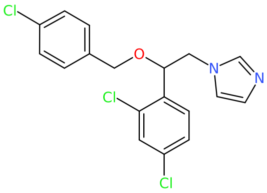 CHEMBL808 IC50:40.0nM						**0.32**							
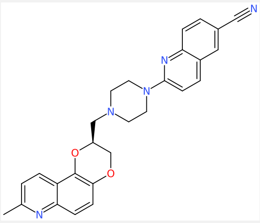 CHEMBL551964 IC50:98.0µM												**0.38**	

structure	mfp1	featmfp1	rdkit7	pattern	ap_bits	tt_bits	fp2	pubchem	cdk_maccs	graph	substructure	hybridization	klekota_roth
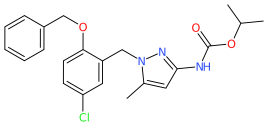 CHEMBL256092 IC50:69.0µM									**0.73**		**0.88**		**0.53**
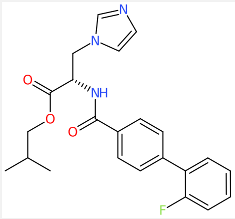 CHEMBL3984743 IC50:1.16µM	**0.47**												

tid: 12911
· chembl id: CHEMBL3397
name: Cytochrome P450 2C9:Homo sapiens

similarity analysis

structure	mfp1	featmfp1	rdkit7	pattern	ap_bits	tt_bits	fp2	pubchem	cdk_maccs	graph	substructure	hybridization	klekota_roth
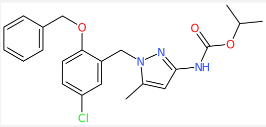 CHEMBL256092 IC50:17.0μM					**0.41**						**0.88**		**0.53**
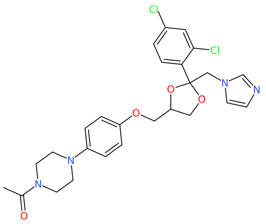 CHEMBL157101 IC50:24.3μM		**0.63**	**0.36**				**0.46**					**0.37**	
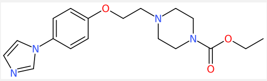 CHEMBL114555 IC50:1.44μM				**0.89**				**0.84**					
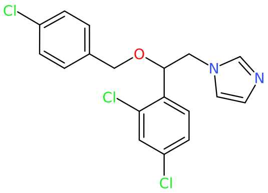 CHEMBL808 IC50:200nM						**0.32**							
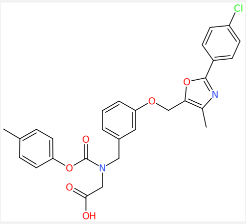 CHEMBL1090211 IC50:3.40μM										**0.55**			

structure	mfp1	featmfp1	rdkit7	pattern	ap_bits	tt_bits	fp2	pubchem	cdk_maccs	graph	substructure	hybridization	klekota_roth
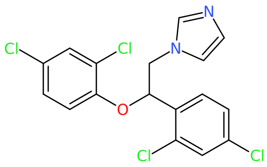 CHEMBL270902 IC50:10.00μM	**0.47**												
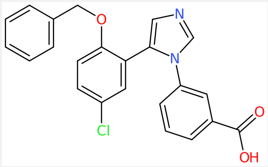 CHEMBL207293 IC50:1.90μM									**0.74**				

tid: 50

chembl id: CHEMBL213

name: Beta-1 adrenergic receptor:Homo sapiens

similarity analysis

structure	mfp1	featmfp1	rdkit7	pattern	ap_bits	tt_bits	fp2	pubchem	cdk_maccs	graph	substructure	hybridization	klekota_roth
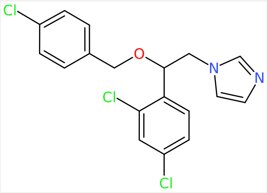 CHEMBL808 IC50:12.8μM					**0.39**								
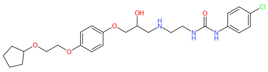 CHEMBL2348202 Kd:11.2nM			**0.38**				**0.44**			**0.61**			
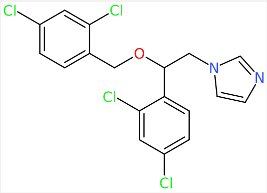 CHEMBL91 IC50:12.6μM		**0.54**									**0.67**		
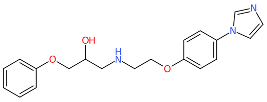 CHEMBL98975 IC50:40.0nM				**0.89**				**0.86**					
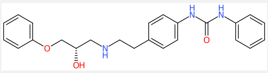 CHEMBL434524 EC50:3.60nM						**0.38**							
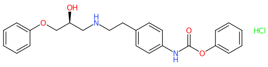 CHEMBL1203992 EC50:40.0nM												**0.47**	**0.39**
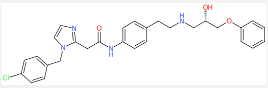 CHEMBL467502 EC50:270nM	**0.55**								**0.72**				

tid: 11365

· chembl id: CHEMBL289

name: Cytochrome P450 2D6:Homo sapiens

similarity analysis

structure	mfp1	featmfp1	rdkit7	pattern	ap_bits	tt_bits	fp2	pubchem	cdk_maccs	graph	substructure	hybridization	klekota_roth
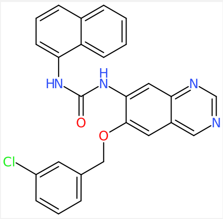 CHEMBL3634101 IC50:5.10μM					0.41						0.75		
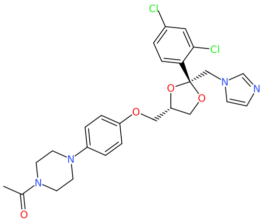 CHEMBL75 IC50:10.0nM		0.63	0.36				0.46						
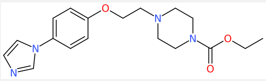 CHEMBL114555 IC50:3.20μM				0.89				0.84					
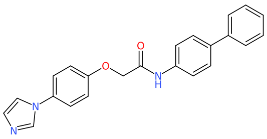 CHEMBL4097104 IC50:100.0nM	0.57					0.40							
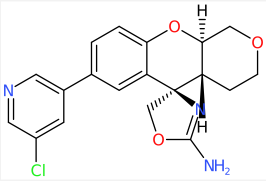 CHEMBL3265335 IC50:1.10μM										0.54			

structure	mfp1	featmfp1	rdkit7	pattern	ap_bits	tt_bits	fp2	pubchem	cdk_maccs	graph	substructure	hybridization	klekota_roth
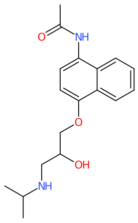 CHEMBL359496 IC50:100.0μM												**0.42**	
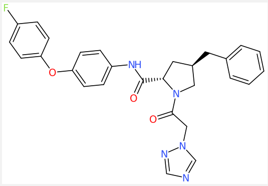 CHEMBL2018484 IC50:392nM									**0.72**				
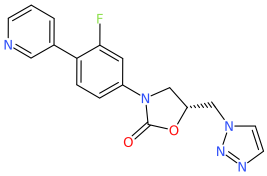 CHEMBL394864 IC50:6.60μM													**0.40**

tid: 20059
chembl id: CHEMBL5035
name: Heme oxygenase 1:Rattus norvegicus

similarity analysis

structure	mfp1	featmfp1	rdkit7	pattern	ap_bits	tt_bits	fp2	pubchem	cdk_maccs	graph	substructure	hybridization	klekota_roth
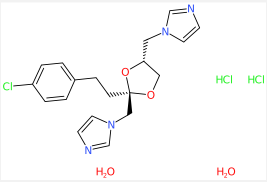 CHEMBL459907 IC50:10.00μM					0.46					0.64			
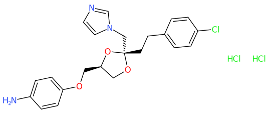 CHEMBL473177 IC50:1.40μM			0.45				0.47	0.84	0.77				
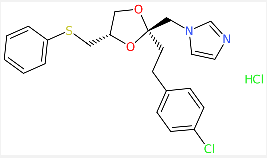 CHEMBL475163 IC50:1.03μM		0.67											
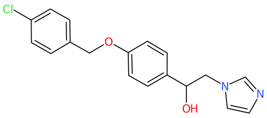 CHEMBL4176027 IC50:13.1μM	0.57			0.85									
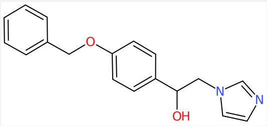 CHEMBL4161217 IC50:500nM						0.42							
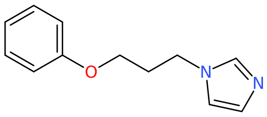 CHEMBL1363752 IC50:31.0μM												0.52	

structure	mfp1	featmfp1	rdkit7	pattern	ap_bits	tt_bits	fp2	pubchem	cdk_maccs	graph	substructure	hybridization	klekota_roth
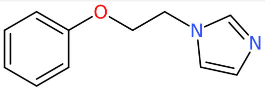 CHEMBL284827 IC50:28.0µM											**0.71**		
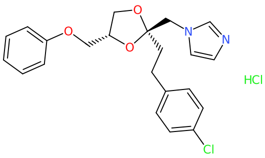 CHEMBL472792 IC50:590nM													**0.42**

tid:	10980
chembl id:	CHEMBL279
name:	Vascular endothelial growth factor receptor 2:Homo sapiens

similarity analysis

structure	mfp1	featmfp1	rdkit7	pattern	ap_bits	tt_bits	fp2	pubchem	cdk_maccs	graph	substructure	hybridization	klekota_roth
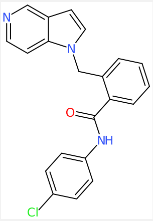 CHEMBL203003 IC50:92.0nM					**0.48**								
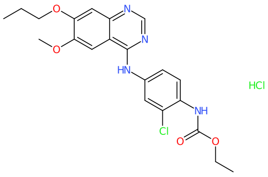 CHEMBL3221547 IC50:500nM			**0.34**				**0.46**						
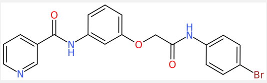 CHEMBL1290247 IC50:5.00nM		**0.65**											
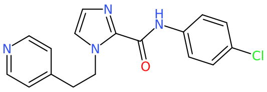 CHEMBL234202 IC50:708nM	**0.53**			**0.78**									
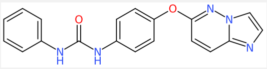 CHEMBL2206661 IC50:5.20μM						**0.33**							
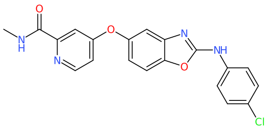 CHEMBL1162958 IC50:766nM										**0.65**			

structure	mfp1	featmfp1	rdkit7	pattern	ap_bits	tt_bits	fp2	pubchem	cdk_maccs	graph	substructure	hybridization	klekota_roth
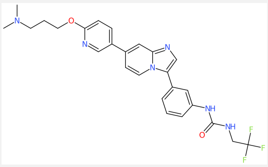 CHEMBL3690500 IC50:35.3nM												0.42	
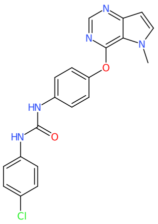 CHEMBL1254752 IC50:32.0nM								0.83					
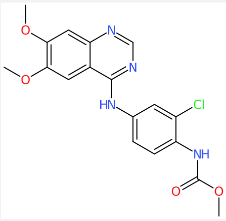 CHEMBL2018751 IC50:5.05µM											0.81		
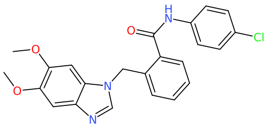 CHEMBL206567 IC50:3.55µM									0.77				
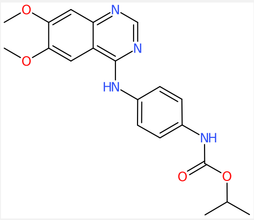 CHEMBL2018750 IC50:5.05µM													0.49

tid: 12887
· chembl id: CHEMBL2289
name: Beta-2 adrenergic receptor:Canis lupus familiaris

similarity analysis

structure	mfp1	featmfp1	rdkit7	pattern	ap_bits	tt_bits	fp2	pubchem	cdk_maccs	graph	substructure	hybridization	klekota_roth
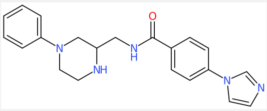 CHEMBL350847 IC50:4.30µM					0.37								
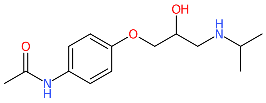 CHEMBL6995 Kd:186nM			0.33				0.41					0.43	
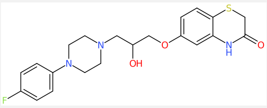 CHEMBL143608 Ki:5.30µM		0.47											
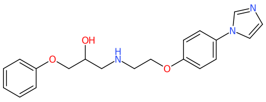 CHEMBL98975 IC50:280nM	0.47			0.89		0.36		0.86	0.66				0.29
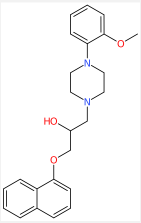 CHEMBL142635 Ki:1.00µM										0.51			
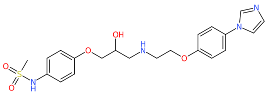 CHEMBL99585 IC50:46.5µM											0.48		

tid: 106616

chembl id: CHEMBL2366472

name: 15-cis-phytoene desaturase:Synechococcus elongatus (strain PCC 7942) (Anacystis nidulans R2)

similarity analysis

structure	mfp1	featmfp1	rdkit7	pattern	ap_bits	tt_bits	fp2	pubchem	cdk_maccs	graph	substructure	hybridization	klekota_roth
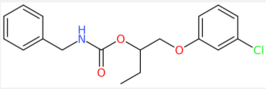 CHEMBL2272986 IC50:50.1nM					0.49								
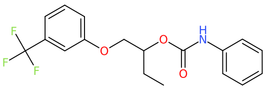 CHEMBL2272975 IC50:50.1nM			0.47			0.46	0.51					0.50	
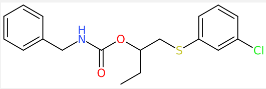 CHEMBL2272700 IC50:5.20μM		0.54											
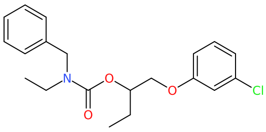 CHEMBL2272703 IC50:63.1nM				0.81					0.64				
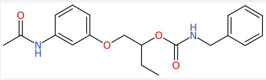 CHEMBL2272713 IC50:3.98μM								0.62		0.58			
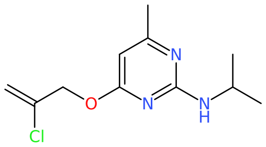 CHEMBL2272974 IC50:7.94μM											0.59		
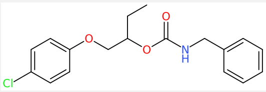 CHEMBL2272987 IC50:3.16μM	0.56												0.65

tid:	261
chembl id:	CHEMBL1811
name:	Prostanoid EP1 receptor:Homo sapiens

similarity analysis

structure	mfp1	featmfp1	rdkit7	pattern	ap_bits	tt_bits	fp2	pubchem	cdk_maccs	graph	substructure	hybridization	klekota_roth
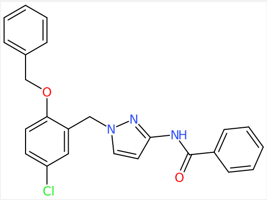 CHEMBL255485 IC50:251nM					**0.46**								
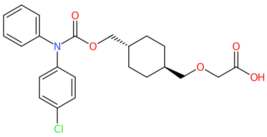 CHEMBL3301604 Ki:9.60µM			**0.36**				**0.46**					**0.36**	
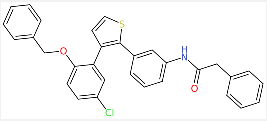 CHEMBL180046 Ki:4.47nM		**0.55**											
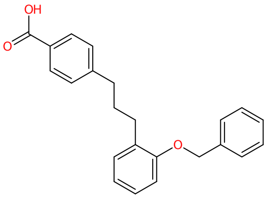 CHEMBL124199 Ki:230nM				**0.71**									
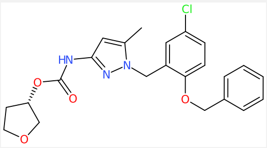 CHEMBL429321 IC50:79.4nM						**0.27**							**0.60**
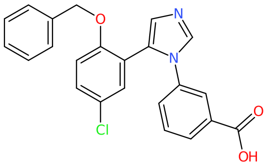 CHEMBL207293 IC50:126nM										**0.53**			

structure	mfp1	featmfp1	rdkit7	pattern	ap_bits	tt_bits	fp2	pubchem	cdk_maccs	graph	substructure	hybridization	klekota_roth
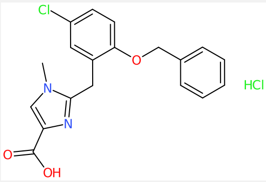 CHEMBL535640 IC50:316nM								0.73					
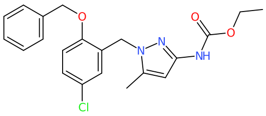 CHEMBL258226 IC50:79.4nM											0.88		
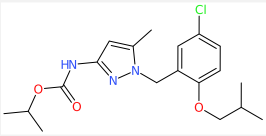 CHEMBL258183 IC50:50.1nM	0.47												
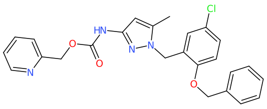 CHEMBL253538 IC50:79.4nM									0.76				

tid:	17045
chembl id:	CHEMBL340
name:	Cytochrome P450 3A4:Homo sapiens

similarity analysis

structure	mfp1	featmfp1	rdkit7	pattern	ap_bits	tt_bits	fp2	pubchem	cdk_maccs	graph	substructure	hybridization	klekota_roth
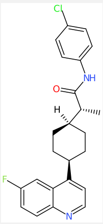 CHEMBL4161733 IC50:5.10μM					0.41								
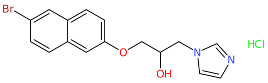 CHEMBL2448692 IC50:700nM			0.50				0.53					0.56	
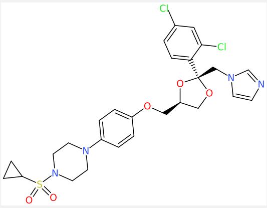 CHEMBL3922888 IC50:185nM		0.66											
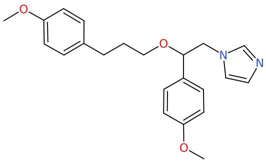 CHEMBL258502 IC50:45.0nM				0.85									
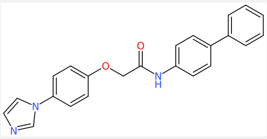 CHEMBL4097104 IC50:20.0nM	0.57					0.40							

structure	mfp1	featmfp1	rdkit7	pattern	ap_bits	tt_bits	fp2	pubchem	cdk_maccs	graph	substructure	hybridization	klekota_roth
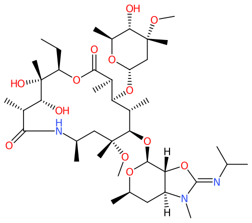<br>CHEMBL2159133<br>IC50:1.00µM										**0.58**			
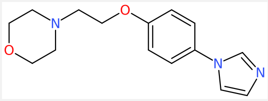<br>CHEMBL113018<br>IC50:5.67µM								**0.82**					
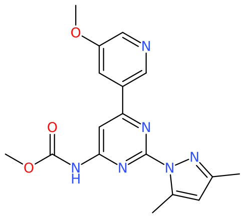<br>CHEMBL519549<br>IC50:16.0µM											**0.81**		
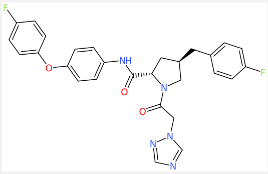<br>CHEMBL2018485<br>IC50:12.6µM									**0.72**				
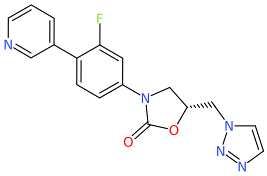<br>CHEMBL394864<br>IC50:6.80µM													**0.40**

tid:	226
chembl id:	CHEMBL246
name:	Beta-3 adrenergic receptor:Homo sapiens

similarity analysis

structure	mfp1	featmfp1	rdkit7	pattern	ap_bits	tt_bits	fp2	pubchem	cdk_maccs	graph	substructure	hybridization	klekota_roth
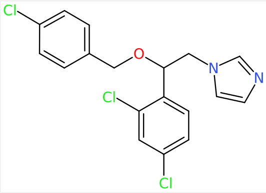 CHEMBL808 IC50:6.15µM					0.39								
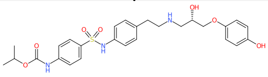 CHEMBL13106 EC50:2.60nM			0.37				0.44						
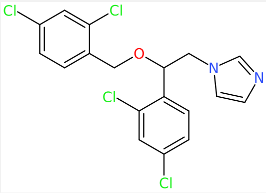 CHEMBL91 IC50:5.48µM		0.54											
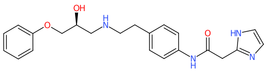 CHEMBL466879 EC50:510nM				0.82									
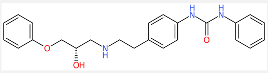 CHEMBL434524 EC50:1.60nM						0.38							
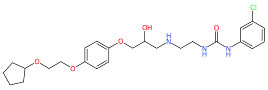 CHEMBL2348201 EC50:10.00µM										0.57			
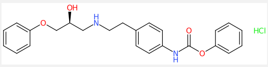 CHEMBL1203992 EC50:2.30µM												0.47	0.39

structure	mfp1	featmfp1	rdkit7	pattern	ap_bits	tt_bits	fp2	pubchem	cdk_maccs	graph	substructure	hybridization	klekota_roth
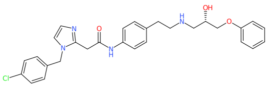 CHEMBL467502 EC50:74.0nM	**0.55**							**0.83**	**0.72**				
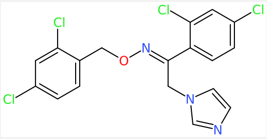 CHEMBL1262 IC50:4.74µM											**0.69**		

tid:	12167
chembl id:	CHEMBL4980
name:	Neuronal acetylcholine receptor protein alpha-7 subunit:Rattus norvegicus

similarity analysis

structure	mfp1	featmfp1	rdkit7	pattern	ap_bits	tt_bits	fp2	pubchem	cdk_maccs	graph	substructure	hybridization	klekota_roth
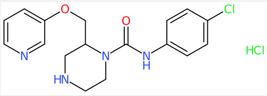 CHEMBL3262153 IC50:70.0nM	0.50				0.52					0.56			
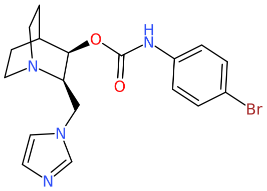 CHEMBL193743 Ki:10.0nM		0.69	0.38			0.34	0.47					0.48	
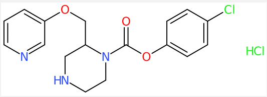 CHEMBL3262155 IC50:9.50nM				0.77					0.73		0.69		
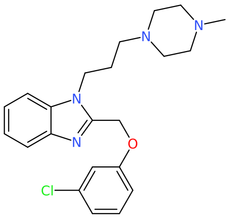 CHEMBL403376 EC50:10.00µM								0.78					
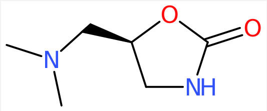 CHEMBL2441754 Ki:94.0µM													0.40

tid:	11280
chembl id:	CHEMBL3227
name:	Metabotropic glutamate receptor 5:Homo sapiens

similarity analysis

structure	mfp1	featmfp1	rdkit7	pattern	ap_bits	tt_bits	fp2	pubchem	cdk_maccs	graph	substructure	hybridization	klekota_roth
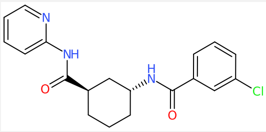 CHEMBL2338561 IC50:5.00µM					**0.43**								
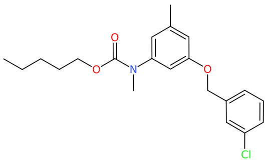 CHEMBL206998 Ki:33.0nM			**0.37**	**0.79**			**0.43**					**0.35**	
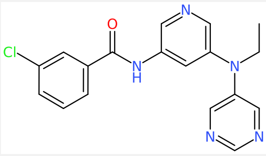 CHEMBL3655656 IC50:260nM		**0.63**											
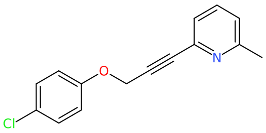 CHEMBL212129 IC50:102nM						**0.25**							
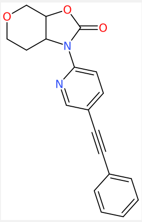 CHEMBL3983913 EC50:60.0nM										**0.58**			

structure	mfp1	featmfp1	rdkit7	pattern	ap_bits	tt_bits	fp2	pubchem	cdk_maccs	graph	substructure	hybridization	klekota_roth
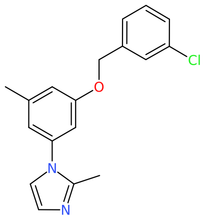 CHEMBL207733 Ki:27.0nM								**0.78**					
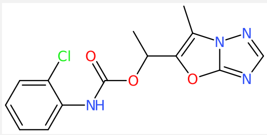 CHEMBL1630060 IC50:501nM											**0.76**		
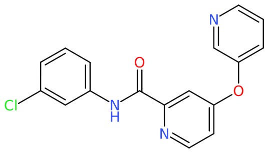 CHEMBL3660209 IC50:390nM	**0.54**												
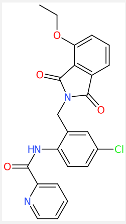 CHEMBL395644 EC50:280nM									**0.71**				
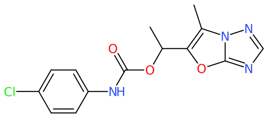 CHEMBL1630062 IC50:2.51µM													**0.66**

tid:	10116
chembl id:	CHEMBL2882
name:	Histamine H2 receptor:Cavia porcellus

similarity analysis

structure	mfp1	featmfp1	rdkit7	pattern	ap_bits	tt_bits	fp2	pubchem	cdk_maccs	graph	substructure	hybridization	klekota_roth
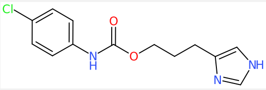 CHEMBL16231 Ki:79.4μM	**0.44**		**0.39**		**0.49**		**0.44**	**0.74**				**0.39**	**0.60**
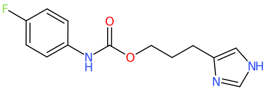 CHEMBL274989 Ki:63.1μM		**0.53**											
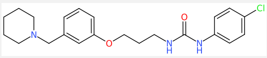 CHEMBL81520 Kd:631nM				**0.76**		**0.30**				**0.51**			
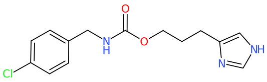 CHEMBL277149 Ki:79.4μM											**0.69**		
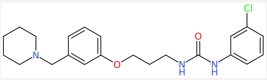 CHEMBL309659 Kd:316nM									**0.65**				

tid: 11300
chembl id: CHEMBL4124
name: Histamine H3 receptor:Rattus norvegicus

similarity analysis

structure	mfp1	featmfp1	rdkit7	pattern	ap_bits	tt_bits	fp2	pubchem	cdk_maccs	graph	substructure	hybridization	klekota_roth
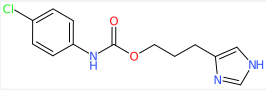 CHEMBL16231 Ki:4.09nM	**0.44**				**0.49**	**0.27**	**0.44**						**0.60**
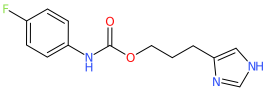 CHEMBL274989 Ki:24.6nM		**0.53**											
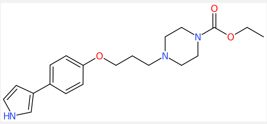 CHEMBL101692 Ki:56.2nM				**0.83**									
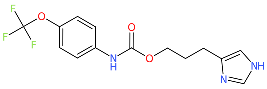 CHEMBL16640 Ki:26.0nM			**0.39**										
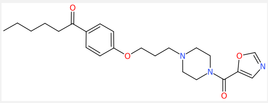 CHEMBL60509 Ki:44.7nM										**0.52**			
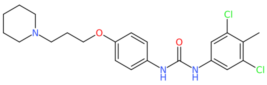 CHEMBL2387307 Ki:135nM												**0.42**	
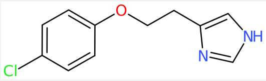 CHEMBL124187 Ki:21.0nM								**0.78**					
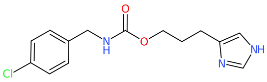 CHEMBL277149 Ki:13.3nM											**0.69**		

structure	mfp1	featmfp1	rdkit7	pattern	ap_bits	tt_bits	fp2	pubchem	cdk_maccs	graph	substructure	hybridization	klekota_roth
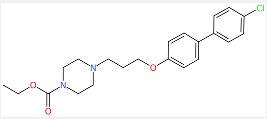 CHEMBL105385 Ki:191nM									**0.68**				

tid:	17074
chembl id:	CHEMBL3943
name:	Histamine H1 receptor:Cavia porcellus

similarity analysis

structure	mfp1	featmfp1	rdkit7	pattern	ap_bits	tt_bits	fp2	pubchem	cdk_maccs	graph	substructure	hybridization	klekota_roth
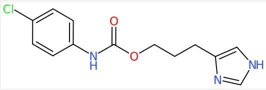 CHEMBL16231 Ki:63.1µM	0.44		0.39		0.49	0.27	0.44					0.39	0.60
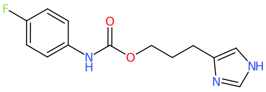 CHEMBL274989 Ki:79.4µM		0.53											
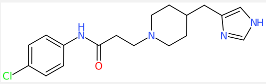 CHEMBL371730 Ki:290nM				0.78									
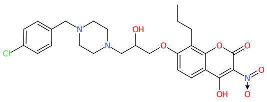 CHEMBL286017 Kd:3.98nM										0.47			
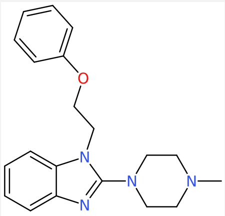 CHEMBL276735 IC50:6.90nM								0.76					
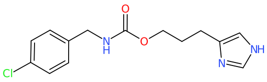 CHEMBL277149 Ki:15.8µM											0.69		
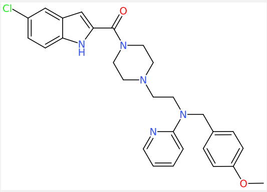 CHEMBL1910379 Kd:20.4nM									0.69				

tid:	103036
chembl id:	CHEMBL1075110
name:	Sterol 14-alpha demethylase:Trypanosoma cruzi (strain CL Brener)

similarity analysis

structure	mfp1	featmfp1	rdkit7	pattern	ap_bits	tt_bits	fp2	pubchem	cdk_maccs	graph	substructure	hybridization	klekota_roth
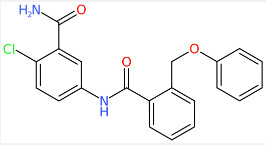 CHEMBL3431227 IC50:79.4µM					0.48								
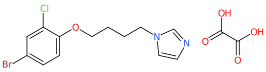 CHEMBL3431397 IC50:63.1nM			0.38				0.45					0.40	
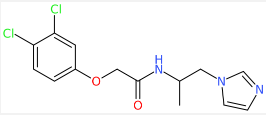 CHEMBL3431305 IC50:63.1nM		0.67							0.69				
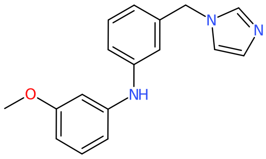 CHEMBL2326052 Kd:210nM				0.78									
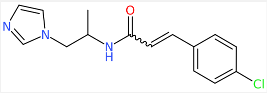 CHEMBL3431250 IC50:158nM						0.36							
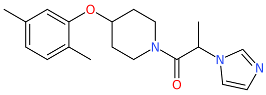 CHEMBL3431307 IC50:100.0nM										0.49			
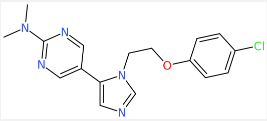 CHEMBL3431242 IC50:50.1nM								0.79			0.79		

structure	mfp1	featmfp1	rdkit7	pattern	ap_bits	tt_bits	fp2	pubchem	cdk_maccs	graph	substructure	hybridization	klekota_roth
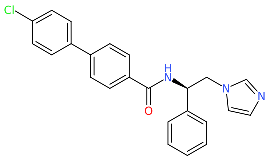 CHEMBL1236676 Kd:70.0nM	**0.58**												
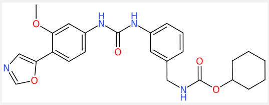 CHEMBL3431205 IC50:25.1μM													**0.40**

tid:	10979
chembl id:	CHEMBL1868
name:	Vascular endothelial growth factor receptor 1:Homo sapiens

similarity analysis

structure	mfp1	featmfp1	rdkit7	pattern	ap_bits	tt_bits	fp2	pubchem	cdk_maccs	graph	substructure	hybridization	klekota_roth
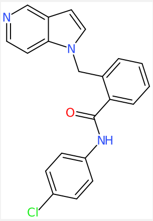 CHEMBL203003 IC50:220nM					0.48								
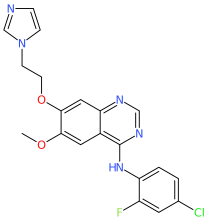 CHEMBL147192 IC50:100.0nM			0.32				0.44	0.75					
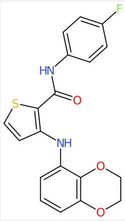 CHEMBL4286029 IC50:1.90µM		0.61											
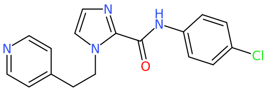 CHEMBL234202 IC50:940nM	0.53			0.78									
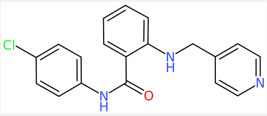 CHEMBL101683 IC50:180nM						0.27							
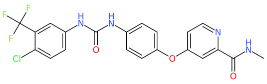 CHEMBL1336 IC50:45.2nM										0.53			

structure	mfp1	featmfp1	rdkit7	pattern	ap_bits	tt_bits	fp2	pubchem	cdk_maccs	graph	substructure	hybridization	klekota_roth
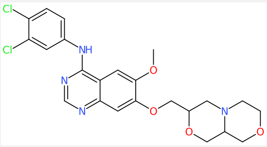 CHEMBL3943570 IC50:50.0nM												**0.36**	
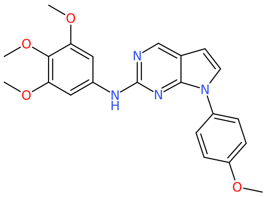 CHEMBL1975927 Ki:251nM											**0.73**		
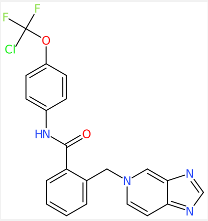 CHEMBL205413 IC50:1.16μM									**0.70**				
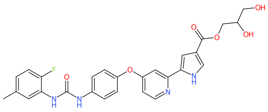 CHEMBL3660290 IC50:16.0nM													**0.41**

tid: 11289

chembl id: CHEMBL3229

name: Anandamide amidohydrolase:Rattus norvegicus

similarity analysis

structure	mfp1	featmfp1	rdkit7	pattern	ap_bits	tt_bits	fp2	pubchem	cdk_maccs	graph	substructure	hybridization	klekota_roth
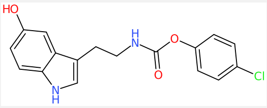 CHEMBL241137 IC50:6.80µM					0.45								
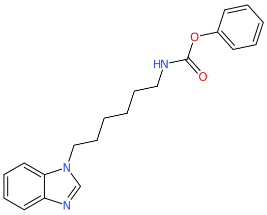 CHEMBL3585522 IC50:290nM				0.80			0.38	0.80					
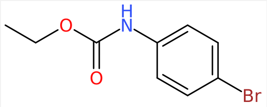 CHEMBL3683934 IC50:4.00µM		0.58											
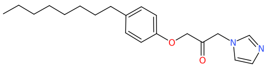 CHEMBL598974 IC50:558nM			0.36									0.42	
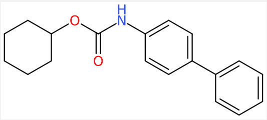 CHEMBL541244 IC50:17.0µM						0.37							0.53
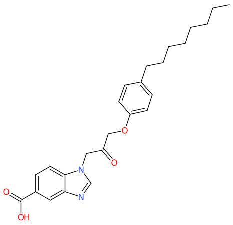 CHEMBL482959 IC50:610nM										0.56			

structure	mfp1	featmfp1	rdkit7	pattern	ap_bits	tt_bits	fp2	pubchem	cdk_maccs	graph	substructure	hybridization	klekota_roth
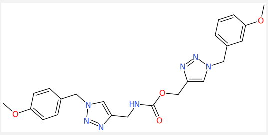 CHEMBL4218592 IC50:5.80nM											**0.93**		
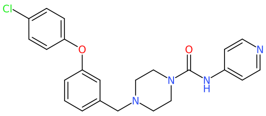 CHEMBL2164601 IC50:520nM	**0.51**												
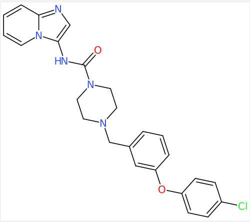 CHEMBL2207351 IC50:119nM									**0.72**				

tid:	12608
chembl id:	CHEMBL4772
name:	1-acylglycerol-3-phosphate O-acyltransferase beta:Homo sapiens

similarity analysis

structure	mfp1	featmfp1	rdkit7	pattern	ap_bits	tt_bits	fp2	pubchem	cdk_maccs	graph	substructure	hybridization	klekota_roth
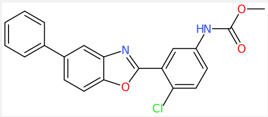 CHEMBL36197 IC50:64.0μM					**0.34**								
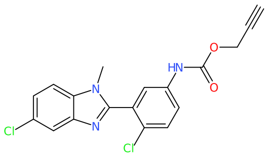 CHEMBL418604 IC50:60.0nM		**0.59**	**0.35**				**0.41**		**0.66**				
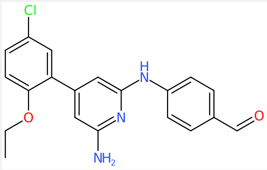 CHEMBL193911 IC50:50.0nM				**0.67**									
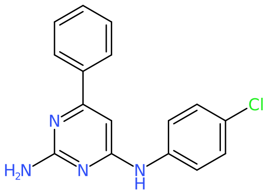 CHEMBL75791 IC50:1.10μM						**0.28**							
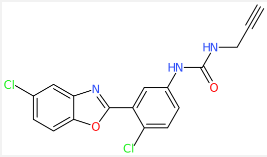 CHEMBL35967 IC50:100.0nM										**0.46**			
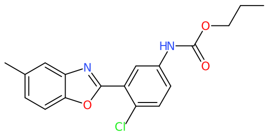 CHEMBL35615 IC50:12.0μM												**0.38**	

structure	mfp1	featmfp1	rdkit7	pattern	ap_bits	tt_bits	fp2	pubchem	cdk_maccs	graph	substructure	hybridization	klekota_roth
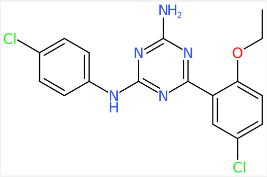 CHEMBL193589 IC50:191nM								0.70					
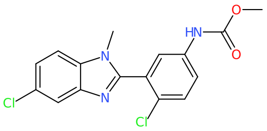 CHEMBL290583 IC50:650nM	0.39										0.87		
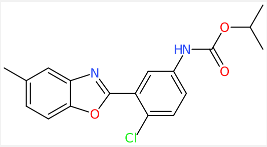 CHEMBL34893 IC50:16.0μM													0.55

tid:	103696
chembl id:	CHEMBL1293263
name:	M18 aspartyl aminopeptidase:Plasmodium falciparum 3D7

similarity analysis

structure	mfp1	featmfp1	rdkit7	pattern	ap_bits	tt_bits	fp2	pubchem	cdk_maccs	graph	substructure	hybridization	klekota_roth
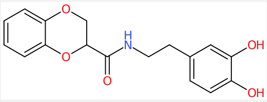 CHEMBL1417867 IC50:2.11µM					**0.35**								
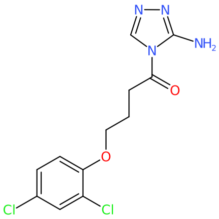 CHEMBL1503953 IC50:2.60µM							**0.36**		**0.68**				
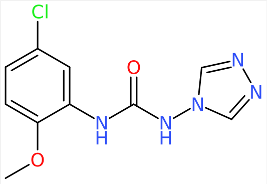 CHEMBL1491018 IC50:832nM		**0.54**	**0.30**					**0.71**			**0.87**	**0.29**	
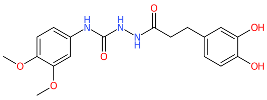 CHEMBL1413985 IC50:2.51µM				**0.68**									
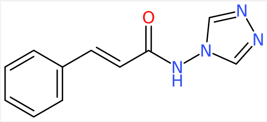 CHEMBL1378749 IC50:1.76µM						**0.22**							

structure	mfp1	featmfp1	rdkit7	pattern	ap_bits	tt_bits	fp2	pubchem	cdk_maccs	graph	substructure	hybridization	klekota_roth
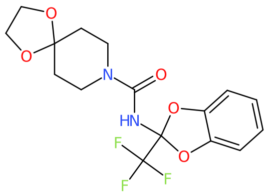 CHEMBL1462474 IC50:2.78μM										**0.44**			
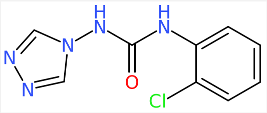 CHEMBL1488151 IC50:1.47μM	**0.39**												
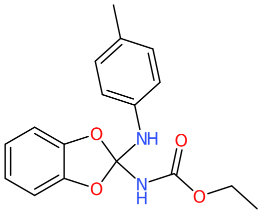 CHEMBL1612425 IC50:5.07μM													**0.40**

tid: 12886
chembl id: CHEMBL2731
name: Galanin receptor 3:Homo sapiens

similarity analysis

structure	mfp1	featmfp1	rdkit7	pattern	ap_bits	tt_bits	fp2	pubchem	cdk_maccs	graph	substructure	hybridization	klekota_roth
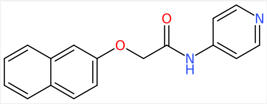 CHEMBL1603921 IC50:1.77µM	**0.50**				**0.44**								
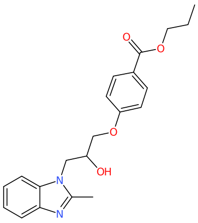 CHEMBL1510261 IC50:1.57µM			**0.40**				**0.48**			**0.56**		**0.50**	
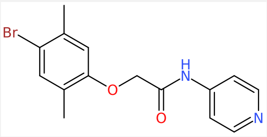 CHEMBL1417355 IC50:1.40µM		**0.65**											
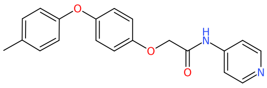 CHEMBL1705098 IC50:158nM				**0.77**									
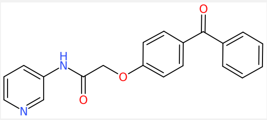 CHEMBL1424583 IC50:399nM						**0.32**							
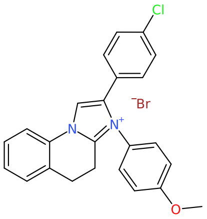 CHEMBL1457690 IC50:5.02µM								**0.74**					

structure	mfp1	featmfp1	rdkit7	pattern	ap_bits	tt_bits	fp2	pubchem	cdk_maccs	graph	substructure	hybridization	klekota_roth
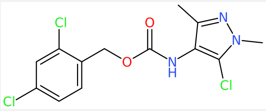 CHEMBL1478769 IC50:2.13μM											0.87		
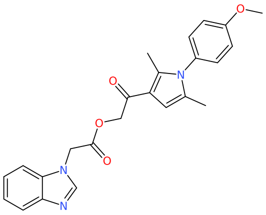 CHEMBL1376266 IC50:762nM									0.66				
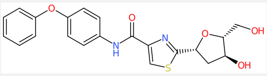 CHEMBL2355112 IC50:400nM													0.40

tid: 10808
· chembl id: CHEMBL3819
name: Lysophosphatidic acid receptor Edg-2:Homo sapiens

similarity analysis

structure	mfp1	featmfp1	rdkit7	pattern	ap_bits	tt_bits	fp2	pubchem	cdk_maccs	graph	substructure	hybridization	klekota_roth
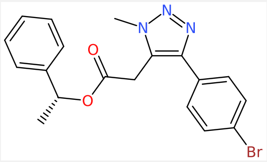 CHEMBL4111515 IC50:21.9µM					0.39								
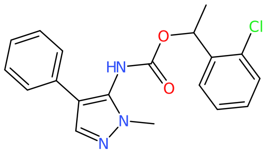 CHEMBL2182056 IC50:3.59µM	0.44						0.30		0.61		0.87	0.29	0.46
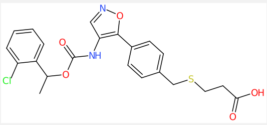 CHEMBL246928 IC50:260nM		0.52											
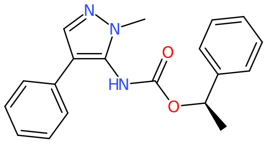 CHEMBL2182053 IC50:17.5µM				0.69									
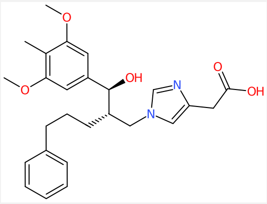 CHEMBL4176330 IC50:180nM			0.32					0.65					
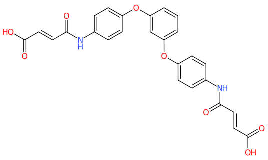 CHEMBL482498 IC50:27.4µM						0.26							

structure	mfp1	featmfp1	rdkit7	pattern	ap_bits	tt_bits	fp2	pubchem	cdk_maccs	graph	substructure	hybridization	klekota_roth
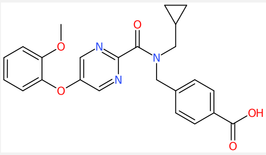 CHEMBL3897650 IC50:170nM										**0.47**			

tid: 10417

· chembl id: CHEMBL4805

name: P2X purinoceptor 7:Homo sapiens

similarity analysis

structure	mfp1	featmfp1	rdkit7	pattern	ap_bits	tt_bits	fp2	pubchem	cdk_maccs	graph	substructure	hybridization	klekota_roth
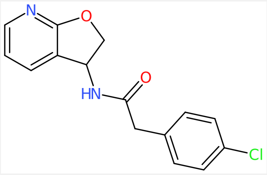 CHEMBL3946649 IC50:1.41µM					**0.44**								
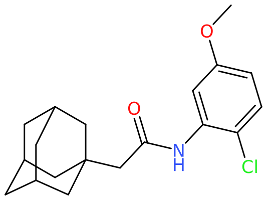 CHEMBL131241 IC50:63.1nM							**0.33**						
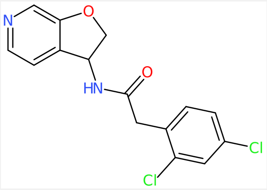 CHEMBL3969023 IC50:15.0nM		**0.54**											
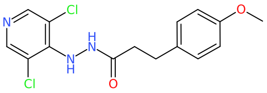 CHEMBL2048264 IC50:1.50µM				**0.71**									
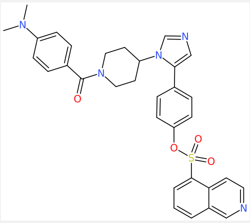 CHEMBL4165149 IC50:0.30nM			**0.31**										

structure	mfp1	featmfp1	rdkit7	pattern	ap_bits	tt_bits	fp2	pubchem	cdk_maccs	graph	substructure	hybridization	klekota_roth
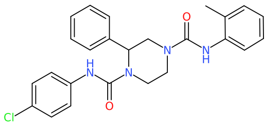 CHEMBL21149 IC50:780nM						**0.23**							
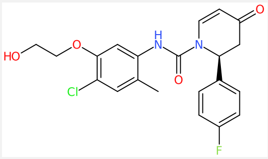 CHEMBL3741412 IC50:9.00nM										**0.51**			
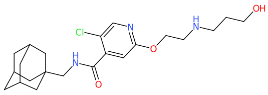 CHEMBL560167 IC50:79.4nM												**0.33**	
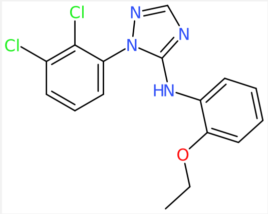 CHEMBL403105 IC50:1.86μM								**0.73**					
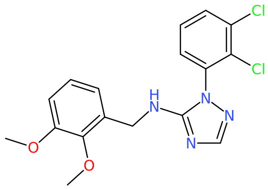 CHEMBL271030 IC50:30.9nM											**0.86**		
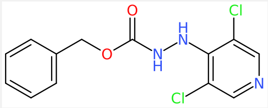 CHEMBL2048274 IC50:370nM	**0.41**												
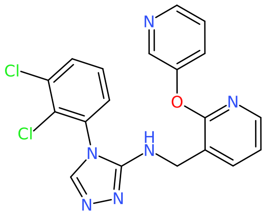 CHEMBL255614 IC50:30.9nM									**0.67**				

structure	mfp1	featmfp1	rdkit7	pattern	ap_bits	tt_bits	fp2	pubchem	cdk_maccs	graph	substructure	hybridization	klekota_roth
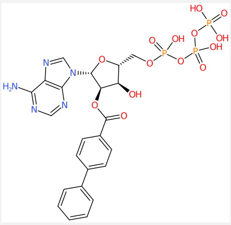 CHEMBL339386 EC50:52.0μM													**0.37**

tid:	10540
chembl id:	CHEMBL2496
name:	P2X purinoceptor 7:Rattus norvegicus

similarity analysis

structure	mfp1	featmfp1	rdkit7	pattern	ap_bits	tt_bits	fp2	pubchem	cdk_maccs	graph	substructure	hybridization	klekota_roth
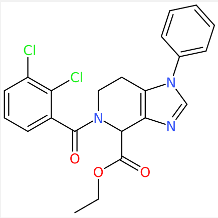 CHEMBL3950348 IC50:1.89μM					**0.43**					**0.47**			
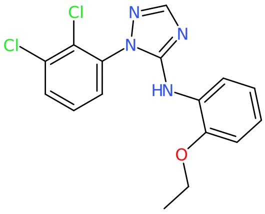 CHEMBL403105 IC50:11.0μM							**0.30**	**0.73**					
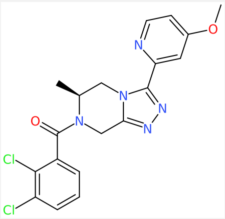 CHEMBL3604426 IC50:10.0nM		**0.45**											
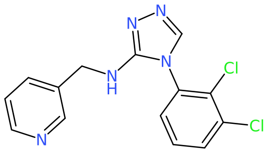 CHEMBL404091 IC50:1.12μM				**0.68**									
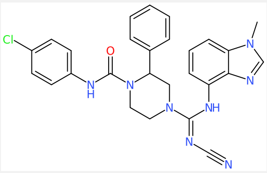 CHEMBL479578 IC50:5.19μM			**0.29**										

structure	mfp1	featmfp1	rdkit7	pattern	ap_bits	tt_bits	fp2	pubchem	cdk_maccs	graph	substructure	hybridization	klekota_roth
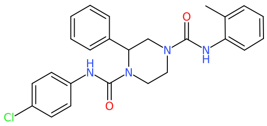 CHEMBL21149 IC50:620nM						**0.23**							
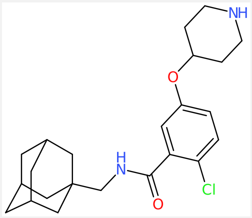 CHEMBL393384 IC50:631nM												**0.32**	
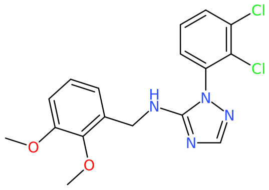 CHEMBL271030 IC50:135nM											**0.86**		
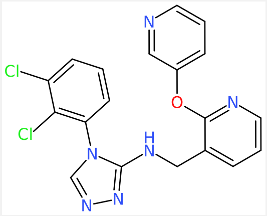 CHEMBL255614 IC50:35.5nM	**0.37**								**0.67**				
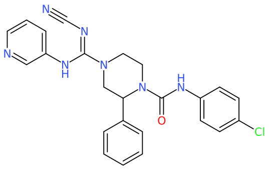 CHEMBL481481 IC50:2.04μM													**0.28**

tid:	102439
chembl id:	CHEMBL6136
name:	Lysine-specific histone demethylase 1:Homo sapiens

similarity analysis

**structure**	mfp1	featmfp1	rdkit7	pattern	ap_bits	tt_bits	fp2	pubchem	cdk_maccs	graph	substructure	hybridization	klekota_roth
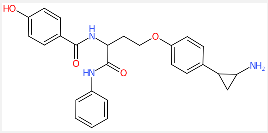 CHEMBL3104339 IC50:1.90µM					**0.43**								
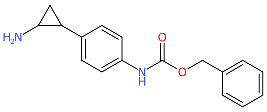 CHEMBL1795981 Ki:1.56µM				**0.74**			**0.34**						**0.38**
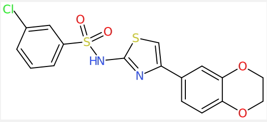 CHEMBL3134368 IC50:29.5µM		**0.54**											
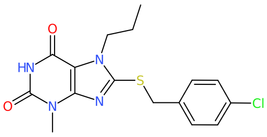 CHEMBL1581095 IC50:25.7µM			**0.28**										
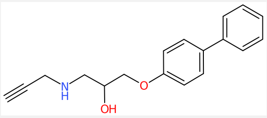 CHEMBL2426048 IC50:38.7µM						**0.32**							
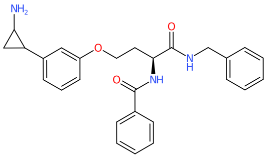 CHEMBL1797639 IC50:2.42µM										**0.43**			

structure	mfp1	featmfp1	rdkit7	pattern	ap_bits	tt_bits	fp2	pubchem	cdk_maccs	graph	substructure	hybridization	klekota_roth
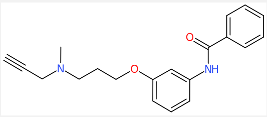 CHEMBL2426047 IC50:93.1μM												**0.36**	
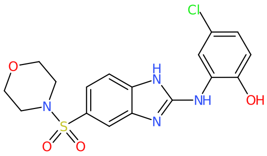 CHEMBL3786908 IC50:90.0μM								**0.66**					
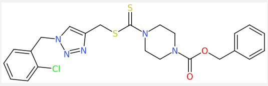 CHEMBL2334486 IC50:53.2μM											**0.86**		
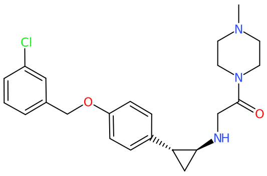 CHEMBL3651589 Ki:47.0nM	**0.34**												
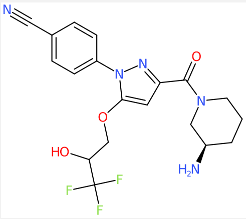 CHEMBL4066743 IC50:155nM									**0.63**				
